# Effects of eating behavior patterns and dietary habits on the therapeutic efficacy of Parkinson’s disease medications: from phenomena, mechanisms to clinical applications

**DOI:** 10.3389/fnut.2026.1969279

**Published:** 2026-10-19

**Authors:** Jingyi Ye, Sarena Jiayao Zhang, Jinzhe Guan, Zi-han Shen, Jin Wang, Ruilin Yang, Muze Huang, Zi-mo Li, Yue Lin, Hengning Gan, Shiyuan Wu, Yujiu Wang, Kaiqiang Yang

**Affiliations:** 1 Department of Cardiovascular Surgery, Shandong Medical and Pharmaceutical University Hospital, Binzhou, China; 2 School of Clinic Medicine, Xiamen Medical College, Xiamen, China; 3 The 1st Medical College of Shandong Medical and Pharmaceutical University, Binzhou, China; 4 International Institute for Integrative Sleep Medicine (WPI-IIIS), University of Tsukuba, Tsukuba, Japan; 5 School of Stomatology, Shandong Medical and Pharmaceutical University, Yantai, China; 6 School of Special Education and Rehabilitation, Shandong Medical and Pharmaceutical University, Yantai, China; 7 School of Basic Medicine, Fujian Medical University, Fuzhou, China; 8 School of Dentistry, Xiamen Medical College, Xiamen, China

**Keywords:** eating behavior patterns, levodopa, Parkinson’s disease, pharmacokinetics, precision nutrition therapy

## Abstract

Parkinson’s disease (PD) is a common progressive neurodegenerative disorder managed primarily through pharmacological dopamine replacement therapies—including levodopa (L-DOPA), dopamine receptor agonists, MAO-B inhibitors, COMT inhibitors, and novel non-dopaminergic agents like adenosine A_2A_ receptor antagonists. However, long-term pharmacotherapy faces major challenges such as motor fluctuations, levodopa-induced dyskinesias, erratic gastrointestinal absorption, and inter-individual response variability. This review systematically synthesizes how dietary habits, macronutrient compositions, dietary patterns (e.g., Western, Eastern, Japanese, Vegetarian, Mediterranean/MIND, Ketogenic), and eating behaviors (such as restrictive eating, compulsive binge eating, and irregular meal timing) serve as critical extrinsic modulators of drug disposition and clinical performance. Multi-dimensional biological mechanisms underpin these interactions: competitive inhibition at LAT1 transporters by dietary large neutral amino acids (LNAAs), food-induced alterations in gastric emptying and hepatic/intestinal enzyme activity (e.g., CYP3A4, CYP1A2, UGT), neuroinflammatory and oxidative stress modulation via nutrient choices, and microbiota–gut–brain axis (MGBA) dynamics involving gut barrier integrity, microbial short-chain fatty acid (SCFA) production, and local bacterial enzymatic degradation of L-DOPA. In real world clinical practice, the absence of precision nutrition frameworks, protein-redistribution diets, targeted chronotherapeutic meal scheduling, and multidisciplinary nutritional support substantially hinders the effective translation of basic pharmacokinetic insights. Consequently, patients frequently encounter formidable clinical challenges, including destabilized pharmacokinetics, recurrent motor complications, and the inability to effectively delay disease progression.

## 1 Introduction

Parkinson’s disease (PD) stands as the second most common chronic and progressive neurodegenerative disorder globally, characterized clinically by cardinal motor features—including resting tremor, rigidity, bradykinesia, and gait impairment—as well as an expansive spectrum of non-motor manifestations such as autonomic dysfunction, cognitive decline, and psychiatric disturbances (1, 2). Driven primarily by global population growth and demographic aging, the burden of PD is surging exponentially across high, middle, and low Socio-Demographic Index (SDI) nations, with the incidence peaking in the eighth decade of life and displaying a marked male preponderance (1). At the neuropathological level, PD is defined by the progressive loss of dopaminergic neurons in the substantia nigra pars compacta coupled with the widespread accumulation of α -synuclein–containing Lewy bodies, an evolving pathological process underpinned by complex interactions between genetic susceptibility and environmental toxicant exposures (2). Recent large scale epidemiological investigations highlight notable regional variations and spatial clustering in PD distribution—exemplified by age-adjusted incidence rates reaching up to 212 per 100,000 in North America among older adults—underscoring the critical need to decipher environmental and lifestyle risk modifiers to inform clinical care delivery and targeted therapeutic strategies (3).

Pharmacological therapy remains the indisputable central pillar in managing PD, primarily utilizing dopamine replacement strategies to restore depleted striatal dopaminergic tone and alleviate cardinal motor symptoms (4). Among them, Levodopa continues to represent the therapeutic gold standard for symptomatic management; however, maintaining long term efficacy is severely constrained by progressive disease advancement and the emergence of severe medication-induced side effects (5). As nigrostriatal neurodegeneration advances into mid-to-late stages, patients frequently experience disabling motor complications, such as motor fluctuations, predictable “wearing-off” phenomena, and levodopa-induced dyskinesias, which substantially impair daily activities and quality of life. These clinical management hurdles are further compounded by the disease’s underlying mechanistic heterogeneity, fluctuating symptom presentation, and unpredictable pharmacokinetic variability, all of which compromise sustained therapeutic drug levels (6). Crucially, despite recent pharmacological advancements and continuous delivery modes, existing drug regimens function predominantly as symptomatic palliatives rather than neuroprotective or pathogenetic interventions capable of arresting neuronal loss. Consequently, optimizing therapeutic outcomes necessitates exploring extrinsic regulatory pathways, including dietary habit modifications and meal scheduling, to stabilize drug disposition and overcome current clinical limitations.

In contemporary clinical pharmacotherapy, patient-specific dietary habits, meal composition, and eating behavior patterns serve as vital modulators of oral drug performance and therapeutic success (7). Concomitant food intake and variable meal schedules alter drug bioavailability through diverse physiological and physical mechanisms, directly influencing the rate and extent of gastrointestinal drug absorption as well as metabolic biotransformation (8). These food–drug interactions may contribute to variability in therapeutic response and therefore represent a potentially modifiable component of PD management. Consequently, a rigorous clinical understanding of dietary contexts, patient adherence, and nutrient-mediated metabolic alterations is imperative for optimizing personalized treatment strategies, and ensuring consistent therapeutic efficacy.

Optimizing pharmacotherapy in PD requires a thorough understanding of oral nutrient interactions that govern drug bioavailability and motor symptom control. This review aims to systematically examine how dietary patterns, meal composition, and eating behaviors influence the efficacy and therapeutic response of anti-Parkinsonian medications, with particular attention to clinically relevant pharmacokinetic mechanisms, treatment response, and potential dietary management strategies. Ultimately, establishing these mechanistic frameworks provides an essential foundation to guide evidence-based dietary strategies and personalized therapeutic protocols, mitigating motor fluctuations and enhancing long-term clinical outcomes for patients with PD (9).

## 2 Pharmacological treatment of PD: current therapies and challenges

### 2.1 Therapeutic effects and limitations of levodopa

Levodopa, the metabolic precursor of dopamine capable of crossing the blood–brain barrier, remains the ultimate “gold standard” and most effective symptomatic therapy for PD, consistently demonstrating superior efficacy over other dopaminergic agents in controlling classic bradykinetic and motor features (10, 11). Upon administration, levodopa substitutes for depleted endogenous transmitter stores in central extrapyramidal dopaminergic pathways, significantly ameliorating motor symptoms, improving daily living activities, and enhancing overall quality of life and age-adjusted life expectancy (12, 13). However, levodopa therapy is heavily constrained by pharmacokinetic (PK) and pharmacodynamic limitations, including delayed and variable gastrointestinal absorption, competition with dietary amino acids for intestinal and blood–brain barrier transport, extensive peripheral metabolism, and a short plasma half-life (~2 h), which collectively yield fluctuating plasma exposure and non-physiological, pulsatile stimulation of striatal dopamine receptors (14).

These pharmacokinetic limitations are route dependent and should not be generalized to all levodopa formulations. Inhaled levodopa bypasses gastrointestinal absorption and can provide rapid intermittent treatment of OFF episodes, although it does not provide continuous dopaminergic delivery. Levodopa-carbidopa intestinal gel (LCIG) infusion bypasses gastric emptying by delivering levodopa directly to the small intestine and can reduce fluctuations in plasma levodopa concentrations, but it still depends on intestinal absorption and requires an invasive delivery system. Continuous subcutaneous levodopa-based delivery, including foslevodopa/foscarbidopa infusion, bypasses the gastrointestinal tract and provides sustained drug delivery, thereby reducing variability associated with oral absorption and improving motor fluctuations. Thus, alternative routes can overcome some, but not all, of the pharmacokinetic limitations of oral levodopa; peripheral metabolism, treatment-related complications, device-related burden, and progression of the underlying disease remain clinically relevant (10, 14).

Consequently, within 5 to 10 years of long-term exposure, 40–80% of patients develop disabling motor complications, such as motor response oscillations (“wearing-off” phenomena) and levodopa-induced dyskinesias (15). Furthermore, levodopa therapy fails to address non-dopaminergic or dopa-resistant features—such as freezing of gait, postural instability, autonomic dysfunction, and cognitive decline—and offers purely symptomatic relief without slowing underlying nigrostriatal neurodegeneration (16).

### 2.2 Therapeutic role and limitations of dopamine receptor agonists

Dopamine receptor agonists (DRAs) represent a fundamental therapeutic class in the pharmacological management of PD, serving as effective initial monotherapy in early-stage disease to delay levodopa initiation and as essential adjunctive therapy in advanced clinical stages. By directly activating postsynaptic striatal dopamine receptors without requiring enzymatic processing by degenerating presynaptic nigrostriatal terminals, DRAs bypass presynaptic metabolic conversion, avoid competition with dietary plasma amino acids (17). Contemporary clinical applications have largely shifted from historical ergoline compounds (such as bromocriptine and pergolide) toward non-ergot agonists (including pramipexole, ropinirole, and rotigotine).

From the perspective of diet–drug interactions, DRAs differ from levodopa because they do not compete with dietary large neutral amino acids for intestinal or blood–brain barrier transport. However, oral formulations such as pramipexole and ropinirole still depend on gastrointestinal transit and absorption; therefore, delayed gastric emptying associated with large or irregular meals may contribute to variability in drug absorption and onset of response. In contrast, transdermal rotigotine bypasses gastrointestinal absorption and is less directly influenced by food intake. Important therapeutic limitations include excessive daytime sleepiness and impulse control disorders, including binge eating, which may further disrupt meal patterns and complicate the consistency of oral drug absorption (18–22). Accordingly, the clinical discussion of DRAs should emphasize route-dependent absorption, treatment tolerability, and eating-behavior-related variability rather than broad receptor pharmacology.

### 2.3 Therapeutic roles and limitations of monoamine oxidase-B (MAO-B) and catechol-O-methyltransferase (COMT) inhibitors

MAO-B and COMT represent two central, distinct enzymatic targets that regulate dopamine metabolism and systemic availability in PD (23).

Clinically, MAO-B inhibitors such as selegiline, rasagiline, and safinamide reduce dopamine degradation but generally provide modest symptomatic efficacy as monotherapy and are frequently used as adjunctive treatment (18–20, 23, 24). Their most relevant dietary consideration is the potential interaction with tyramine-rich foods; older irreversible agents therefore warrant caution with high-tyramine products such as aged cheese and wine (18–20, 23, 24). COMT inhibitors, including entacapone, opicapone, and tolcapone, are likewise used mainly as adjuncts to levodopa because inhibition of levodopa methylation prolongs its systemic exposure (24, 26). Their therapeutic limitations include an increased risk of levodopa-related dyskinesia and, for tolcapone, potential hepatotoxicity. Co-administration with meals may modestly alter the bioavailability of specific formulations, whereas the major diet-related clinical consequence of COMT inhibition is its effect on levodopa pharmacokinetics rather than a broad independent food interaction (18, 24, 26). Thus, dietary counseling should focus on clinically relevant food restrictions, meal-related variability, and the way these adjuncts modify levodopa exposure.

### 2.4 Therapeutic role and limitations of adenosine A2A receptor antagonists and other novel drugs

Targeting non-dopaminergic pathways via adenosine $A_{2A}$ receptor antagonists represents a significant milestone in expanding the pharmacological armamentarium for PD, particularly in mitigating motor fluctuations and off-time (25). Although this approach can reduce OFF time, variable inter-individual efficacy and dyskinesia remain important clinical limitations.

Among this class, istradefylline is clinically used as an adjunct to levodopa to reduce OFF time (26–29). In the context of this review, the dietary relevance of A2A receptor antagonism should be presented cautiously. Dietary caffeine is itself a non-selective adenosine receptor antagonist and may have competitive or synergistic interactions with A2A-targeted therapy; however, the current evidence summarized in this manuscript does not establish a robust, clinically actionable food–drug interaction (26–29). Therefore, this section should emphasize the limited and still uncertain dietary evidence for A2A antagonists, together with variability in therapeutic response and dyskinesia risk, rather than providing an extensive overview of investigational receptor pharmacology.

### 2.5 Factors influencing drug absorption, metabolism, and therapeutic response

Systemic bioavailability and subsequent therapeutic response of orally administered medications are determined by an interplay of dynamic physiological, biochemical, and genetic variables. Upon ingestion, drug absorption primarily takes place across the small intestine, a process heavily dictated by gastrointestinal transit times, local mucosal perfusion, gastric pH, and formulation-specific dissolution profiles (30). Physiological alterations—such as delayed gastric emptying, malabsorption syndromes, or surgical resections of the proximal gut—significantly impede or delay absorption kinetics, precipitating sub-therapeutic plasma concentrations or erratic therapeutic windows (31). Following absorption, biotransformation pathways in the intestine and liver govern the extent of pre-systemic first-pass metabolism, which is catalyzed predominantly by the cytochrome P450 (CYP) enzyme superfamily and regulated by drug efflux transporters like P-glycoprotein (32). Inter-individual variability in clinical efficacy and toxicity is fundamentally driven by pharmacogenetic polymorphisms within these drug-metabolizing enzymes and transport proteins, yielding distinct metabolic phenotypes ranging from poor to ultrarapid metabolizers (33). Furthermore, extrinsic influences—including concurrent drug interactions, patient age, liver function, and dietary habits—frequently alter enzyme expression and activity, ultimately modulating systemic clearance, plasma protein binding, and drug concentration at the target effector site (26). A summary of the pharmacological characteristics, clinical challenges, and food/dietary interactions of major PD medications is presented in Table 1. The pharmacological characteristics, clinical challenges, and modulating dietary factors across major PD medication classes are systematically illustrated in Figure 1.

**Table 1:** Summary of pharmacological characteristics and food-drug interactions of commonly used PD medications.

Drug class	Representative agents	Core mechanism of action	Major clinical challenges/limitations	Primary food/dietary interactions	References
Levodopa	Levodopa (+ Carbidopa/Benserazide)	Dopamine precursor; crosses the blood–brain barrier to be converted into dopamine	Short half-life; long-term use leads to motor fluctuations (“wearing-off”) and dyskinesia	High-protein diets (LNAA competition for absorption); delayed gastric emptying from high-fat meals	(13–17, 23, 24)
Dopamine Receptor Agonists (DRAs)	Pramipexole, Ropinirole, Rotigotine	Directly activates postsynaptic striatal dopamine receptors	Impulse control disorders (ICDs, e.g., binge eating), excessive daytime sleepiness	Binge eating induces delayed gastric emptying, indirectly impairing absorption; no competition with dietary amino acids	(18–20)
MAO-B Inhibitors	Selegiline, Rasagiline, Safinamide	Selectively inhibits central dopamine degradation by MAO-B enzyme	Modest symptomatic efficacy as monotherapy; mostly used as adjuncts	Older irreversible agents require caution with high-tyramine foods (e.g., aged cheese/wine) due to the “cheese effect”	(26, 25, 30, 31)
COMT Inhibitors	Entacapone, Opicapone, Tolcapone	Block peripheral or central COMT to extend levodopa plasma half-life	Increased risk of levodopa -related dyskinesia; potential hepatotoxicity (e.g., Tolcapone)	Co-administration with meals may slightly alter bioavailability for specific formulations; primary effect is optimizing levodopa kinetics	(25, 32, 33)
Adenosine A2A Antagonists	Istradefylline	Modulates non-dopaminergic basal ganglia pathways to reduce “OFF” time	Variable inter-individual efficacy; may exacerbate dyskinesia	Dietary caffeine (a non-selective A2A antagonist) exhibits potential competitive or synergistic interaction	(26–29)

**Figure 1:**
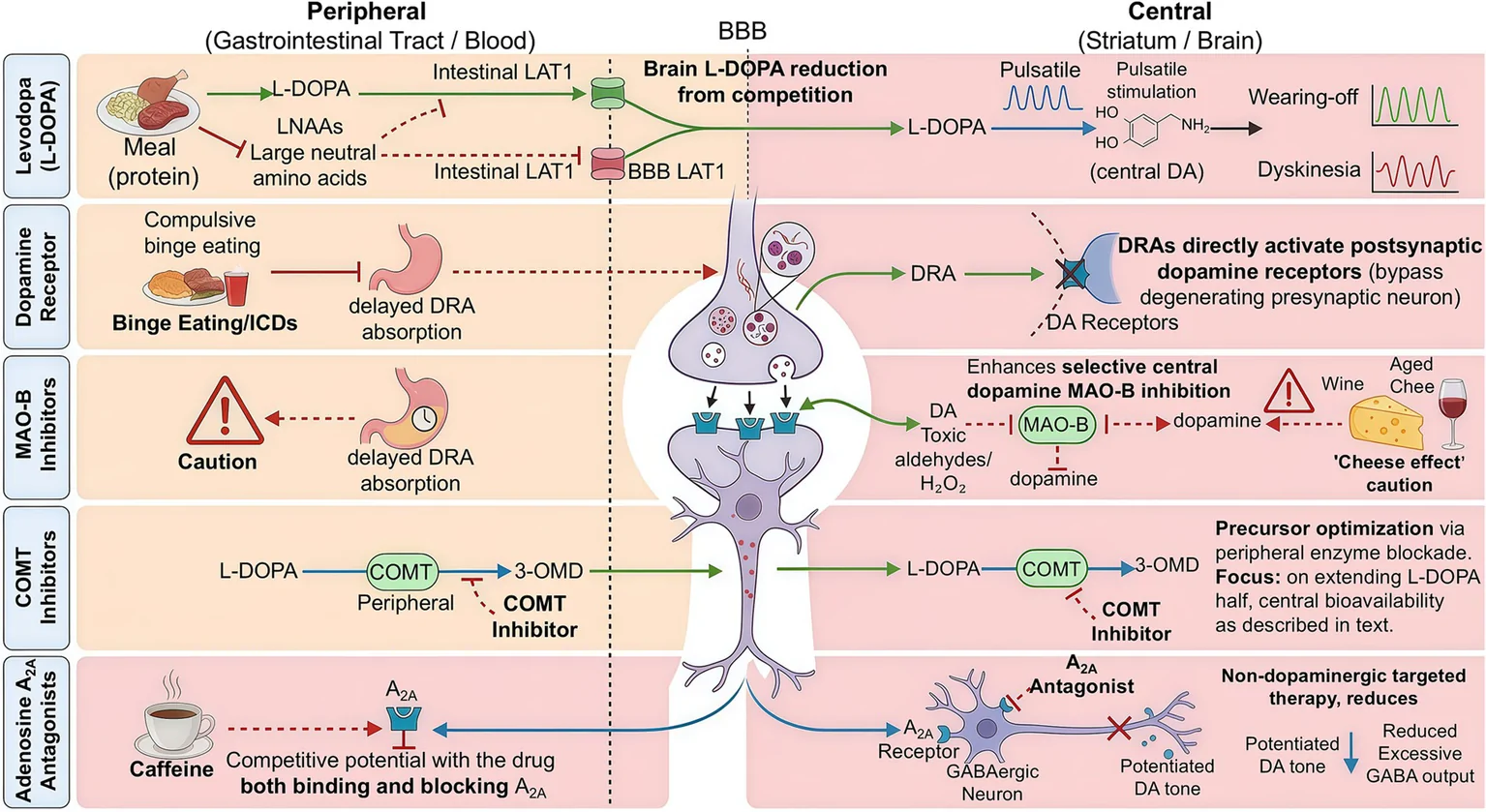
Pharmacological spectrum, pharmacokinetic/pharmacodynamic limitations, and extrinsic dietary modulators of primary PD medications. This schematic illustrates the distinct mechanistic targets and clinical challenges associated with primary dopaminergic and non-dopaminergic therapies in PD. The therapeutic cascade initiates with levodopa, the principal dopamine precursor, which is fundamentally constrained by a short plasma half-life and severe competitive inhibition at intestinal and blood–brain barrier transport sites by dietary large neutral amino acids (LNAAs). Concurrently, postsynaptic dopamine receptor agonists (DRAs) directly activate striatal dopamine receptors to bypass presynaptic conversion, yet their systemic bioavailability remains highly susceptible to delayed gastric emptying induced by compensatory binge eating behaviors. The local metabolic microenvironment is further regulated by metabolic enzyme inhibitors (MAO-B and COMT), which extend dopaminergic tone but are subject to dietary interactions, such as the “cheese effect” associated with high-tyramine foods in older agents. Finally, non-dopaminergic targeted agents, specifically adenosine A2A receptor antagonists like istradefylline, modulate GABAergic striatopallidal pathways to reduce “off” time, a pathway that exhibits potential competitive or synergistic interaction with dietary caffeine.

## 3 Interactions between dietary patterns, eating behaviors, and anti-parkinsonian treatments

### 3.1 Impact of the westernized diet on levodopa bioavailability, systemic inflammation, and pharmacological outcomes

Westernized dietary patterns, characterized by excessive consumption of saturated fats, ultra-processed foods, and refined sugars, may influence factors relevant to levodopa response; however, direct clinical evidence that a Westernized dietary pattern as a whole reduces the efficacy of anti-Parkinsonian medications remains limited. Chronic intake of high-fat and high-sugar foods reshapes the gastrointestinal architecture and disrupts gut microbiota homeostasis, fostering a pro-inflammatory microbial environment and escalating systemic low-grade inflammation (27). These observations support potential biological pathways through which components of a Westernized diet could influence treatment response, but they do not by themselves demonstrate reduced levodopa efficacy in patients. Furthermore, high-fat dietary intake may alter gastrointestinal transit and could thereby contribute to variability in the absorption of orally administered levodopa, although direct evidence linking a Westernized dietary pattern to reduced levodopa exposure is limited (28). Such dysbiosis and altered motility may be associated with small intestinal bacterial overgrowth, while commensal bacteria expressing tyrosine decarboxylase can metabolize levodopa into peripheral dopamine before mucosal uptake (34). Accordingly, bacterial levodopa metabolism represents a plausible mechanism for reduced systemic levodopa availability; however, the proposed sequence linking Western diet-induced dysbiosis, increased bacterial levodopa metabolism, and poorer therapeutic response remains largely indirect (29). Among specific dietary components, high protein intake has more direct evidence for affecting the clinical response to levodopa because large neutral amino acids derived from dietary protein compete with levodopa for transport across the intestinal epithelium and blood–brain barrier, which can contribute to delayed “on” states and “wearing-off” phenomena (8). High-fat meals may influence levodopa response primarily through effects on gastric emptying and gastrointestinal transit rather than through competition at large neutral amino acid transporters (28). Collectively, current evidence more strongly supports specific dietary components, particularly high protein intake, as clinically relevant modifiers of levodopa response than it supports a direct effect of the Westernized dietary pattern as a whole. Microbiota-mediated pathways are biologically plausible but should be interpreted as indirect mechanisms until they are confirmed by prospective clinical studies specifically evaluating medication response in patients with PD. Direct clinical studies in PD provide stronger evidence for specific meal-related interventions than for broad regional dietary patterns. In particular, low-protein or protein-redistribution strategies and meal timing have been shown to alter levodopa clinical response or pharmacokinetics in susceptible patients. These direct human studies are summarized separately in Supplementary Table S1 to distinguish established medication-related evidence from indirect mechanistic hypotheses.

### 3.2 Neuroprotective and pharmacokinetic benefits of the Mediterranean diet in PD pharmacotherapy

Current evidence does not establish that adherence to the Mediterranean diet (MeDi) modifies the course of PD or enhances the efficacy of anti-Parkinsonian pharmacotherapy. Adherence to the Mediterranean diet (MeDi) has been associated with favorable neurological and metabolic outcomes in PD, but direct evidence that MeDi improves the efficacy or pharmacokinetics of specific anti-Parkinsonian medications remains limited. Several original human studies provide more limited support for potential clinical relevance: higher MeDi adherence has been associated with lower odds of PD, later age at onset, and lower prodromal PD probability, while small randomized trials in patients with PD have reported improvements in disease-severity or cognitive outcomes after short-term MeDi interventions (21, 22, 35, 36). These findings should be interpreted as associative or short-term clinical evidence rather than proof of disease modification or improved levodopa pharmacokinetics. Mechanistic literature suggests that components of the Mediterranean diet may influence oxidative stress, neuroinflammatory signaling, and neuronal survival pathways; however, these mechanistic observations should not be interpreted as evidence that MeDi modifies PD progression in patients (37). In the longitudinal study by Maraki et al., higher MeDi adherence was associated with a smaller increase in prodromal PD probability over approximately 3 years and with a lower incidence of possible/probable prodromal PD and PD/dementia with Lewy bodies among 1,047 older adults without PD or dementia with Lewy bodies at baseline (22). These findings should be regarded as preliminary observational evidence and require confirmation in other populations. They do not establish a causal or disease-modifying effect of MeDi, nor do they demonstrate improved responsiveness to dopaminergic therapy. The MED-PARK study is a randomized controlled trial protocol designed to evaluate the effects of a modified Mediterranean diet on motor and non-motor symptoms, with immune, metabolomic, and microbiome outcomes as secondary measures (40). Because this publication is a protocol, it provides no efficacy results or conclusions and is cited only to indicate an ongoing investigation rather than as evidence of clinical benefit or altered anti-Parkinsonian medication requirements.

### 3.3 Interactions between traditional east Asian diets and anti-Parkinsonian medications

Traditional East Asian dietary components may interact with mechanisms relevant to anti-Parkinsonian pharmacotherapy, but the strength of evidence varies across individual components and is often preclinical. For soy protein, the available evidence is largely mechanistic or preclinical; for instance, soy protein combined with time-restricted feeding suppresses monoamine oxidase B (MAO-B) activity, preserves dopaminergic neurons, and mitigates neuroinflammation while maintaining motor function in Parkinsonian models (38). However, because dietary proteins yield large neutral amino acids that compete with levodopa for intestinal and blood–brain barrier transport mechanisms, precise dietary timing remains critical. Bioactive polyphenols abundant in green tea, such as epigallocatechin-3-gallate (EGCG), act as multitarget agents capable of inhibiting MAO-B enzymatic activity and preventing α -synuclein aggregation, although direct clinical evidence that EGCG improves the response to dopaminergic medication remains limited (39). Nevertheless, high concentrations of green tea catechins can competitively inhibit intestinal uptake transporters such as organic anion transporting polypeptides (OATP1A2/OATP2B1), potentially altering systemic drug exposure and pharmacokinetic parameters (34). Complementing terrestrial dietary constituents, marine-derived compounds like omega-3 polyunsaturated fatty acids serve as valuable adjuncts by reducing neuroinflammation and ameliorating non-motor symptoms like depression, without impairing levodopa pharmacokinetics (40). Furthermore, traditional herbal formulations such as Zhengan Xifeng Decoction significantly increase levodopa peak concentration ( $C_{\max}$ ) and overall area under the curve ( ${\mathrm{AUC}}_{0-\infty }$ ), prolonging systemic half-life while reducing medication-associated adverse events. The cited Zhengan Xifeng Decoction study provides a more direct pharmacokinetic example, although its preclinical design limits clinical generalization. Thus, this subsection distinguishes direct pharmacokinetic observations from mechanistic or preclinical interactions and avoids generalizing them as established clinical effects on anti-Parkinsonian treatment (41).

### 3.4 Effects of dietary fat, protein load, and meal timing on levodopa pharmacokinetics and clinical response

Dietary fat, protein load, and meal timing may influence oral levodopa disposition through effects on gastric emptying, gastrointestinal transit, and amino-acid competition for drug transport.

High caloric density and elevated lipid content significantly prolong gastric emptying time by inducing shallow peristalsis and enhancing antral retention, which delays the intestinal transfer of co-administered therapeutic agents (42, 43). For oral levodopa, direct human evidence is stronger for protein load and meal timing. Randomized and crossover studies have reported poorer motor response with high-protein intake and improvement with low-protein or protein-redistribution strategies in susceptible patients (44–46). Meal timing is also clinically important: in patients receiving controlled-release levodopa/carbidopa, postprandial dosing delayed levodopa appearance and Tmax, reduced the 6-h AUC, and delayed motor-response onset compared with premeal dosing (47). The principal effects of dietary fat, protein intake, and meal timing on gastrointestinal transit, levodopa absorption, and clinical response are summarized in Figure 2.

**Figure 2:**
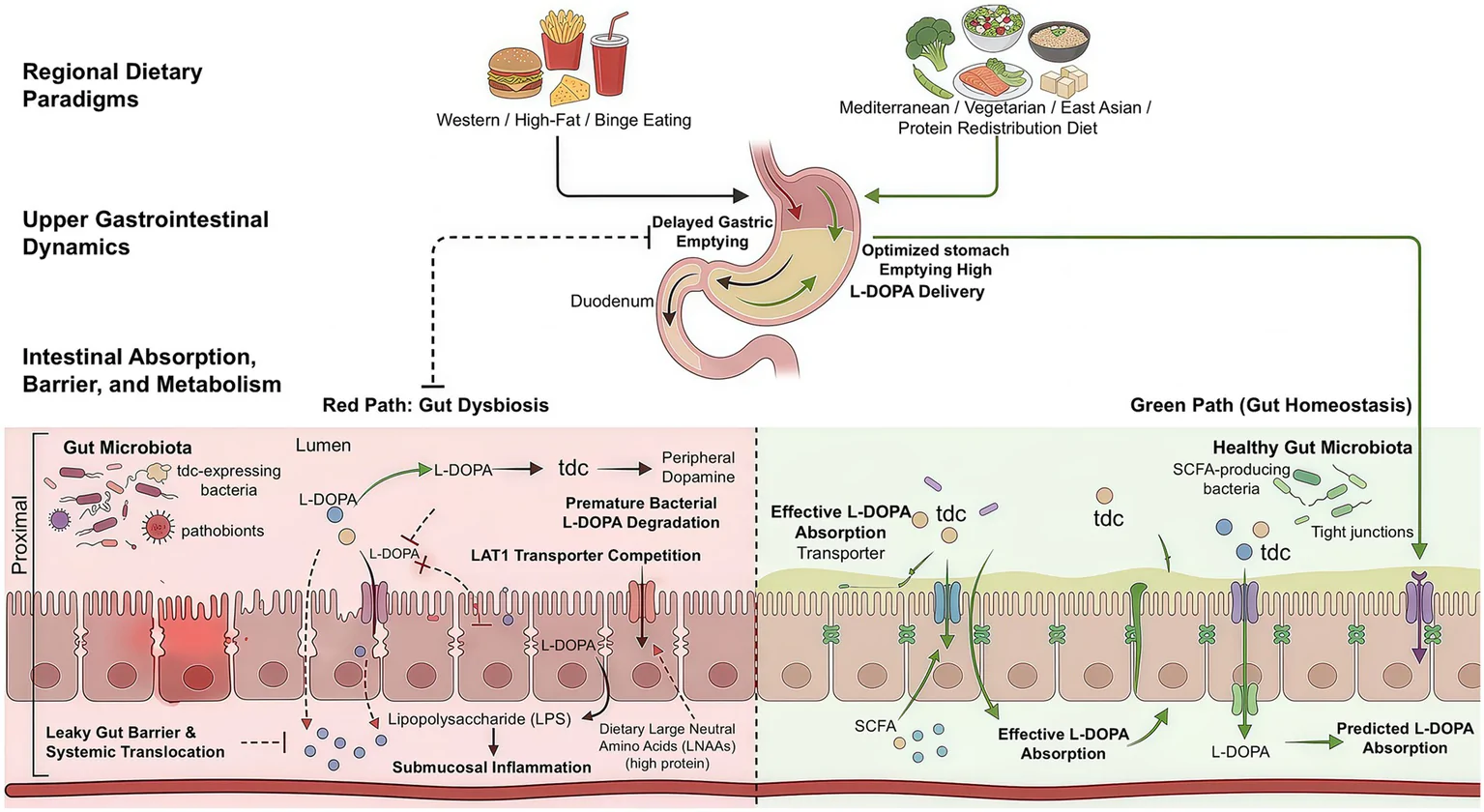
Spatial mapping and pharmacokinetic interactions of PD pharmacotherapy across distinct regional dietary frameworks. This visual maps the disparate impacts of macroscopic regional dietary structures on systemic drug disposition and neuroprotection. High-fat, animal-protein-rich Northern diets induce shallow peristalsis and prolonged antral retention, severely delaying gastric emptying while generating intense LNAA competition that precipitates sudden “wearing-off” motor fluctuations. In stark contrast, the Mediterranean diet operates as a neuroprotective and pharmacokinetic stabilizer, utilizing monounsaturated fatty acids and polyphenols to preserve the intestinal mucosal barrier, thereby suppressing systemic microglial activation and optimizing predictable drug absorption. Traditional East Asian dietary patterns exhibit a complex dual-modulatory role; bioactive compounds such as epigallocatechin-3-gallate (EGCG) from green tea synergistically inhibit MAO-B enzymatic activity, while elevated soy protein intake necessitates rigorous protein-redistribution scheduling to avert LAT1 transport competition.

### 3.5 Traditional African dietary patterns, fiber-rich carbohydrates, and potential modulatory roles in PD therapy

Traditional African dietary patterns are often fiber-rich and may support intestinal microbial diversity and mucosal function, but direct medication-specific evidence in PD is limited (48). The complex dietary fibers and resistant starches inherent to these indigenous staple foods act as vital fermentable substrates for the intestinal microbiota, preserving gut lining integrity, promoting host immune tolerance, and downregulating pro-inflammatory signaling pathways (49). Within the context of PD pathophysiology, progressive gastrointestinal motility dysfunction and gut dysbiosis frequently compromise the integrity of the intestinal epithelial barrier, aggravating systemic neuroinflammation and impeding optimal pharmacotherapeutic absorption. Targeted nutritional restoration using prebiotic fibers and complex carbohydrate matrices can favorably rebalance the host gut microbiome, enhancing short-chain fatty acid biosynthesis while dampening the neuroinflammatory cascades implicated in disease progression (50). Clinically, intervention with resistant starch—a major functional component of traditional high-fiber diets—has been demonstrated to selectively enrich beneficial taxa such as *Faecalibacterium* species, attenuate pro-inflammatory proteomic markers, and significantly alleviate clinical motor and non-motor symptoms in PD cohorts. These findings may support gastrointestinal or microbiome-oriented adjunctive care, but they should not be presented as evidence of improved anti-Parkinsonian drug efficacy unless medication exposure or treatment-response outcomes are measured directly.

### 3.6 Legume- and maize-based Latin American diets: protein competition and pharmacokinetic dynamics

Diets featuring a heavy reliance on starchy legumes (*Phaseolus vulgaris*) and maize (*Zea mays*) provide a high concentration of plant proteins and large neutral amino acids (LNAAs) that significantly influence systemic amino acid pools and drug absorption kinetics (51). Traditional Latin American staple diets typically combine maize—which is inherently low in lysine and tryptophan but rich in leucine—with legumes to achieve essential amino acid complementation (52). Protein-derived LNAAs can compete with levodopa for transport across the intestinal epithelium and blood–brain barrier; however, direct evidence that legume- and maize-based Latin American dietary patterns specifically alter levodopa pharmacokinetics or motor response is lacking (53). Moreover, anti-nutritional factors present in starchy legumes, such as enzyme inhibitors and phytic acid, reduce overall protein digestibility and lead to erratic post-prandial amino acid bioavailability. Clinical studies indicate that higher dietary protein intake can reduce levodopa responsiveness in susceptible patients, whereas low-protein or protein-redistribution strategies may improve motor response (44–46). The distinct intestinal absorption cascades, microbial fermentative pathways, and LAT1 transporter competition across South Asian, Southeast Asian, African, and Latin American dietary paradigms are systematically detailed in Figure 3.

**Figure 3:**
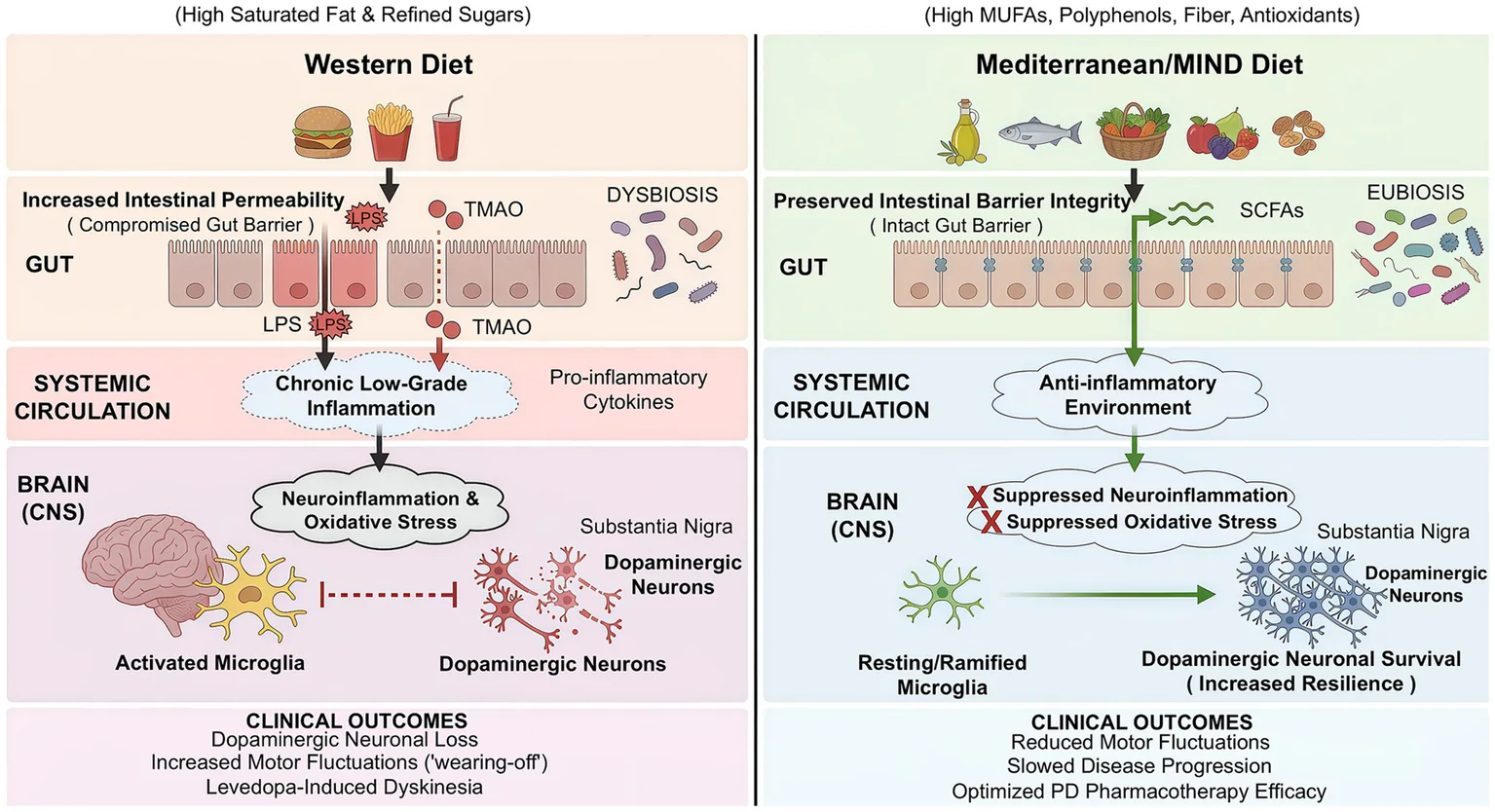
Mechanistic impact of tropical, spiced, fiber-dense, and starchy staple dietary paradigms on drug disposition, microbiota dynamics, and LAT1 competition. This figure delineates the specific pharmacokinetic alterations driven by South Asian, Southeast Asian, African, and Latin American dietary paradigms. In Southeast Asian diets, complex botanical mixtures and crude plant extracts (e.g., *Ocimum sanctum*) actively modulate phase I biotransformation by directly inhibiting major hepatic and intestinal cytochrome P450 enzymes like CYP3A4. Traditional African diets, characterized by unrefined fiber-rich carbohydrates and resistant starches, act as vital fermentable substrates that selectively enrich beneficial taxa such as *Faecalibacterium*, subsequently increasing short-chain fatty acid (SCFA) biosynthesis to downregulate neuroinflammatory signaling. Conversely, Latin American staple diets reliant on starchy legumes and maize flood the gastrointestinal tract with a high concentration of plant-derived LNAAs, directly impairing both the peripheral pharmacokinetics and central nervous system uptake of levodopa via saturable LAT1 carrier competition.

### 3.7 Comparative mechanistic synthesis and global clinical considerations for regional dietary management in PD

The strength of evidence for diet-treatment interactions in PD is uneven. Direct human evidence is concentrated on oral levodopa and specific modifiable factors, particularly protein quantity, protein distribution across the day, meal composition, and meal timing relative to dosing. In contrast, evidence for broader regional or dietary patterns—including Mediterranean, Western, East Asian, African, Latin American, plant-rich vegetarian, and Southeast Asian dietary contexts—is derived predominantly from studies of PD risk, symptoms, disease progression, gut microbiota, inflammation, or general pharmacology rather than from direct measurements of anti-Parkinsonian drug exposure or efficacy.

Plant-rich, high-carbohydrate dietary patterns may alter intestinal transit and gut microbial composition, providing plausible mechanisms through which diet could influence oral drug disposition. Likewise, botanical products and fermented foods may modulate drug-metabolizing enzymes or intestinal transporters. However, these observations are largely mechanistic or extrapolated from general pharmacological studies, and current evidence does not establish that vegetarian, spiced, botanical-rich, fermented-food, or Southeast Asian dietary patterns as a whole produce clinically meaningful changes in the pharmacokinetics or therapeutic efficacy of specific anti-Parkinsonian medications. Accordingly, these factors should be regarded as hypothesis-generating mechanistic considerations rather than established diet–drug interactions (40, 54–63).

By comparison, clinical evidence more consistently supports the effects of protein-related LNAA competition and meal timing on levodopa response. Lower or redistributed protein intake can improve ON time or motor response in protein-sensitive patients, whereas the timing of meals relative to levodopa administration can alter drug absorption and the onset of clinical response (44–47). These observations suggest that clinically relevant dietary management should focus primarily on specific nutritional components and medication–meal relationships rather than on broad geographical dietary labels.

Accordingly, dietary recommendations should be individualized according to medication formulation, protein sensitivity, nutritional status, gastrointestinal function, and observed motor response. The comparative effects of regional and functional dietary patterns on drug bioavailability, the microbiota–gut–brain axis, and clinical outcomes are summarized in Table 2 as a mechanistic overview, whereas Supplementary Table S1 summarizes direct human evidence linking dietary factors or meal timing to levodopa pharmacokinetics or clinical response. The principal gastrointestinal mechanisms—including delayed gastric emptying, altered intestinal arrival, and competition between levodopa and dietary LNAAs for transport—are illustrated in Figure 4, while Figure 5 summarizes microbiota–gut–brain axis pathways that may indirectly influence medication disposition and PD-related outcomes.

**Table 2:** Comparative analysis of global dietary patterns on pharmacokinetics, MGBA, and PD progression.

Dietary pattern	Key nutritional characteristics	Impact on drug bioavailability & pharmacokinetics	Impact on gut microbiota & systemic inflammation	Clinical outcome & disease progression	References
Western Diet	High in saturated fats, ultra-processed foods, refined sugars, low fiber	Delays gastric emptying; promotes bacterial tyrosine decarboxylase (tdc) expression, increasing peripheral levodopa breakdown	Induces dysbiosis, compromises gut barrier integrity, increases LPS translocation and neuroinflammation	Erratic plasma levodopa levels, frequent “OFF” periods, accelerated neurodegeneration	(8, 34, 38–40)
Mediterranean/MIND Diet	High in MUFAs, polyphenols, antioxidant vitamins, dietary fibers	Preserves gut mucosal barrier; optimizes predictable drug absorption profiles	Enriches SCFA-producing bacteria, lowers TMAO, suppresses systemic and microglial activation	Reduced motor fluctuations, attenuated neurodegenerative progression, improved non-motor symptoms	(41, 54–57)
East Asian Diet	Rich in green tea polyphenols (EGCG), soy proteins, marine omega-3 s	EGCG inhibits MAO-B; high soy intake requires protein-redistribution timing to prevent LAT1 competition	Restores intestinal microbiota balance; suppresses gut-derived pro-inflammatory signals	Synergistic neuroprotective effects; improved motor stability with timed protein intake	(40, 43, 58–63)
High-Protein/Animal-Fat Northern Diet	Calorie-dense, elevated saturated lipids and animal proteins	Severe LNAA competition at intestinal mucosa and BBB LAT1 carriers; delayed gastric transit	Increases bile acid metabolizing bacteria, alters gut transit dynamics	Marked “wearing-off” fluctuations, delayed “ON” response time	(50, 51, 53, 109)
Fiber-Rich/Plant-Based Vegetarian Diet	High microbial-accessible carbohydrates, complex fibers, spices	Accelerates intestinal transit; minimizes amino acid competition if plant proteins are balanced	Enrich *Bifidobacterium* and *Prevotella*; increases SCFA production and mucosal barrier strength	Stabilizes motor performance, reduces constipation and GI dysmotility complications	(42–46)

**Figure 4:**
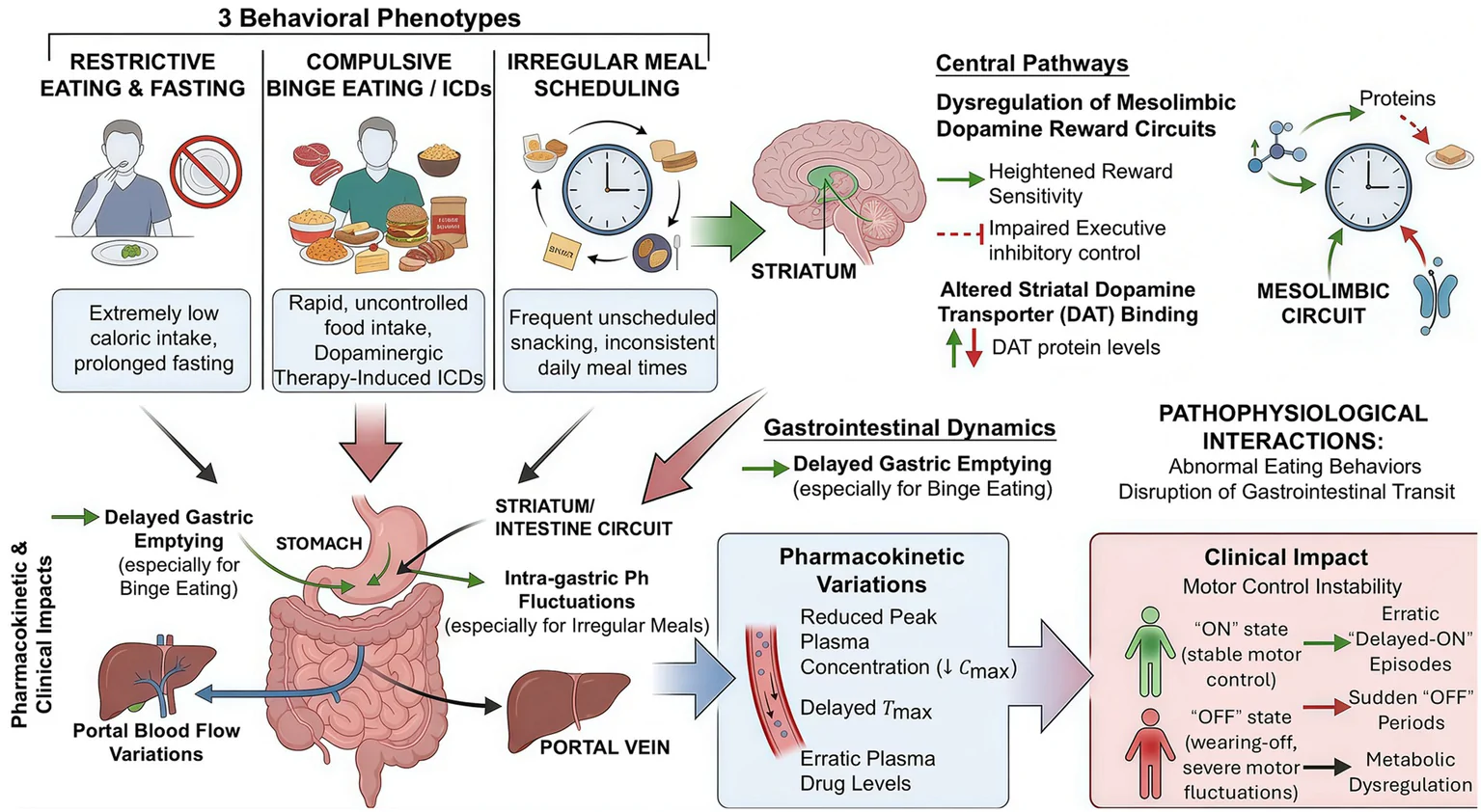
Luminal gastrointestinal mechanisms dictating oral antiparkinsonian drug absorption. This schematic details the fine-scale physiological and micro-environmental cascades occurring within the upper gastrointestinal tract following drug ingestion. Upon administration, the systemic bioavailability of therapeutic agents is heavily dictated by gastric motility and luminal fluid dynamics; high-lipid dietary loads significantly prolong gastric emptying time by enhancing antral retention, thereby hindering the rapid duodenal transfer required for time-sensitive absorption. Once within the proximal small intestine, the absorption cascade is further bottlenecked by competitive saturation at the enterocyte mucosal layer. Here, dietary protein digestion products directly compete with exogenous levodopa for access to the L-type amino acid transporter 1 (LAT1) system, a process dynamically influenced by concurrent fluctuations in gastric pH, local microvascular splanchnic perfusion, and intestinal transit times.

**Figure 5:**
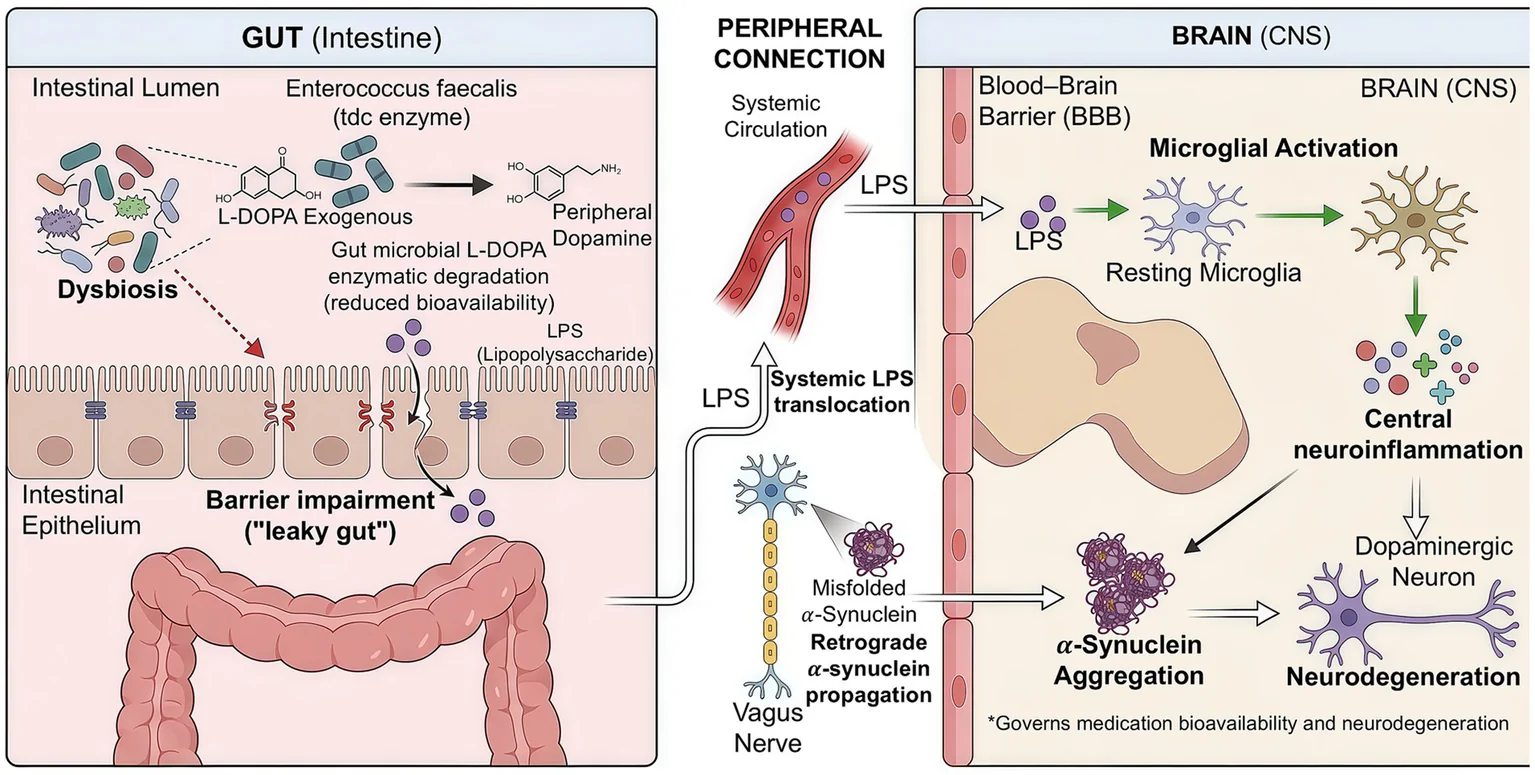
Microbiota–gut–brain axis (MGBA) mechanisms modulating PD medication efficacy. This illustration captures the bidirectional interplay between host intestinal dysbiosis, local drug metabolism, and central neuroinflammation. Driven frequently by high-fat and ultra-processed Western diets, the microbial ecosystem shifts toward a pro-inflammatory state, facilitating the proliferation of commensal bacteria that express tyrosine decarboxylase (tdc). This local bacterial enzymatic activity orchestrates the premature presystemic degradation of levodopa into peripheral dopamine prior to mucosal uptake, drastically narrowing the therapeutic window. Concurrently, this dysbiotic environment compromises gut mucosal barrier integrity, promoting the systemic translocation of lipopolysaccharides (LPS) and pro-inflammatory metabolites. These molecular triggers subsequently propagate retrograde neuroinflammatory signaling via the vagus nerve, establishing a gut-brain feedback loop that exacerbates drug resistance and clinical motor instability.

Beyond dietary composition, eating behaviors can further modify anti-Parkinsonian treatment through changes in nutritional intake, meal size, gastrointestinal transit, and medication-meal timing. The following subsection therefore integrates the major behavior-related modifiers of treatment response, including restrictive eating, binge eating, and medication–meal timing.

### 3.8 Eating behaviors, meal timing, and clinical implications for PD pharmacotherapy

Restrictive eating, binge eating, and irregular meal timing may influence anti-Parkinsonian treatment through changes in nutritional status, gastrointestinal transit, meal composition, and the temporal relationship between food intake and medication administration. Restrictive eating can alter body composition and metabolic state; however, much of the evidence regarding calorie restriction is general or preclinical and does not establish improved pharmacokinetics or efficacy of specific anti-Parkinsonian medications. For oral levodopa, the clinically relevant distinction is between unstructured dietary restriction, which may compromise nutritional adequacy, and structured manipulation of protein intake or meal timing, for which direct treatment-related evidence is stronger (44–47).

Binge eating is also clinically relevant because dopaminergic therapy, particularly dopamine agonists, can precipitate impulse-control disorders that include compulsive eating (60, 61). Large or protein-rich meals during such episodes may delay gastric emptying and increase competition between dietary large neutral amino acids and levodopa, thereby contributing to variability in levodopa absorption and motor response (62). Accordingly, management should address both treatment-related eating behaviors and potentially modifiable meal characteristics when these factors coincide with motor fluctuations.

Meal timing represents one of the better-supported behavioral determinants of oral levodopa response. In patients receiving controlled-release levodopa/carbidopa, postprandial dosing delayed levodopa appearance and Tmax, reduced the 6-h AUC, and delayed the onset of motor response compared with premeal administration (47). Protein-rich meals may further exacerbate LNAA competition, making the timing of levodopa administration particularly relevant in protein-sensitive patients (44–46). Nevertheless, medication–meal schedules should be individualized according to levodopa formulation, nutritional status, gastrointestinal function, and observed clinical response rather than applying a universal fasting strategy.

More broadly, abnormal eating behaviors in PD may arise from disease-related feeding difficulties, dopaminergic treatment-related impulse-control disorders, or irregular meal patterns. Reduced intake and dysphagia may contribute to weight loss and malnutrition, whereas hyperphagia and compulsive eating may be triggered or exacerbated by dopaminergic therapy (63–70). These factors can affect adherence, meal composition, and medication timing and may indirectly destabilize treatment response. Routine screening and multidisciplinary management are therefore appropriate when abnormal eating behavior coexists with motor fluctuations, substantial weight change, malnutrition risk, or impulse-control symptoms. Table 3 summarizes these treatment-relevant eating behaviors and management considerations, while Figure 6 illustrates their relationships with gastrointestinal transit, drug exposure, and motor control.

**Table 3:** Impact of eating behavior patterns and meal timing on PD therapy and clinical strategies.

Eating behavior/pattern	Characteristic features	Physiological & pharmacokinetic (PK) mechanism	Impact on drug efficacy & clinical outcomes	Clinical management strategies	References
Restrictive Eating/Calorie Restriction	Extremely low caloric intake, prolonged fasting, or strict dietary restriction	Shifts body composition, alters CYP3A4/UGT enzymes, and accelerates serotonin clearance via monoamine transporters	Reduces central drug efficacy; while it promotes autophagy, unguided restriction risks malnutrition	Avoid unmonitored severe fasting; implement structured intermittent fasting under clinical guidance	(63–68)
Binge Eating/Compulsive Eating	Rapid consumption of large, dense meals (often secondary to dopamine agonist therapy)	Acute heavy intake severely delays gastric emptying and alters hepatic CYP3A4 and UGT enzyme clearance	Causes erratic drug absorption, triggering unexpected “OFF” periods, delayed “ON” responses, and severe motor fluctuations	Screen for Impulse Control Disorders (ICDs); adjust dopamine agonist dosage accordingly	(47, 69–72)
Irregular Meal Timing & High Frequency	Frequent unscheduled snacking and inconsistent daily meal times	Causes fluctuating gastric pH, erratic gastric emptying, and altered splanchnic blood flow	Leads to unpredictable plasma drug concentrations, increasing intra- and inter-individual PK variability	Establish a standardized, consistent daily meal schedule	(73–76)

**Figure 6:**
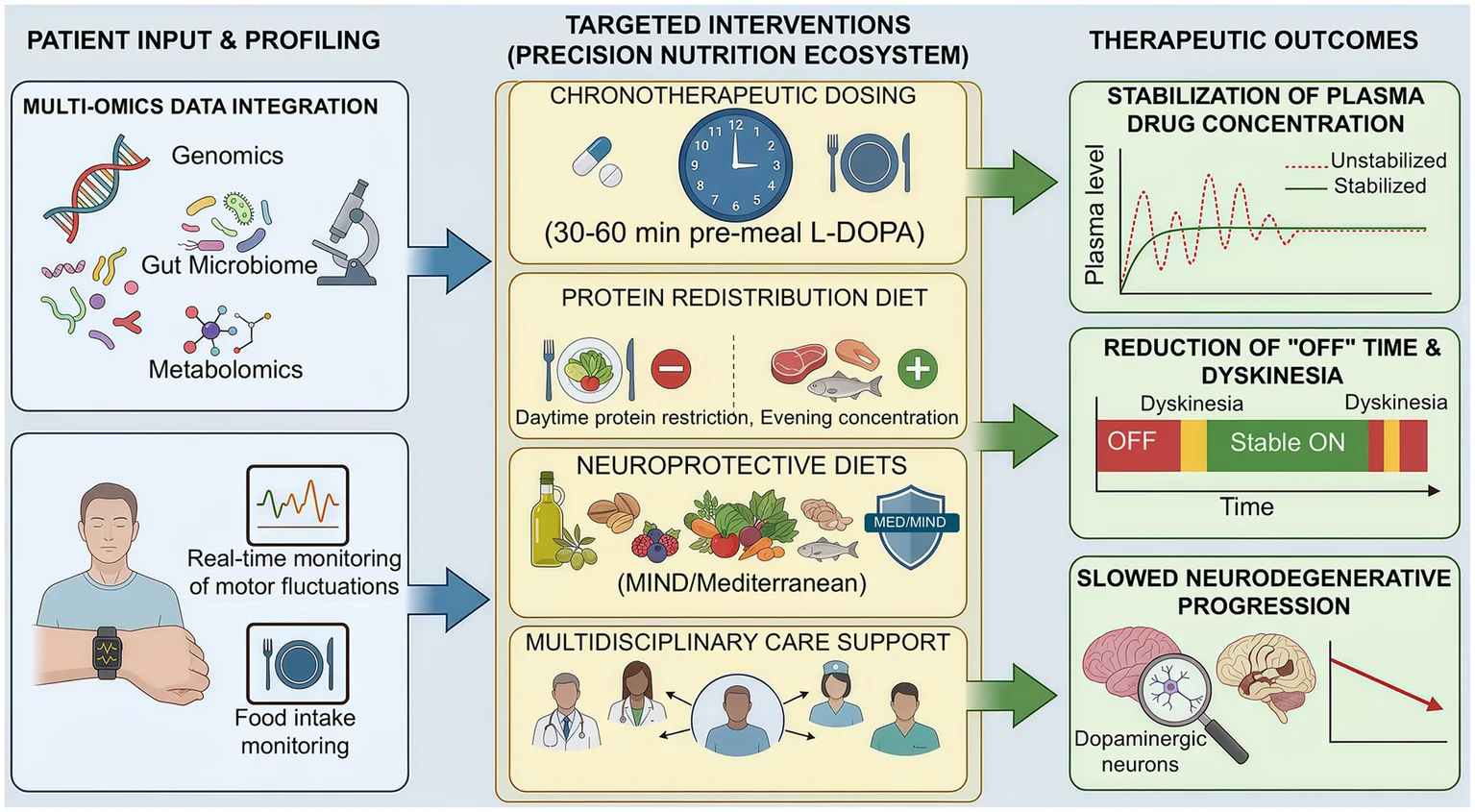
Neurobiological, metabolic, and pharmacokinetic cascades of abnormal eating behavior patterns in PD. This framework highlights how aberrant, disease- or medication-driven eating behaviors destabilize systemic drug disposition. Restrictive eating and severe caloric deprivation induce fundamental metabolic shifts, altering hepatic CYP3A4 and UDP-glucuronosyltransferase (UGT) clearance activities while accelerating central serotonin clearance via monoamine transporter adaptations. In contrast, compulsive binge eating—frequently emerging as an impulse control disorder (ICD) secondary to dopamine receptor agonist therapy—triggers massive macronutrient overloads. This rapid intake severely delays gastric emptying and floods the system with competitive dietary amino acids, causing erratic drug absorption, delayed “on” state transitions, and severe postprandial reactive hypoglycemia. Furthermore, irregular meal scheduling and unscheduled high-frequency snacking continuously disrupt baseline gastric pH and splanchnic blood flow, introducing profound intra-individual pharmacokinetic variability.

## 4 Mechanisms underlying the effects of dietary factors on PD drug efficacy

### 4.1 Regulatory effects of dietary factors on levodopa pharmacokinetics

The regulatory management of dietary factors plays a pivotal role in modulating levodopa pharmacokinetics, directly influencing its systemic absorption and cerebral bioavailability in patients with PD. Because levodopa utilizes the saturable large neutral amino acid (LNAA) transport system across both the intestinal mucosa and the blood–brain barrier (BBB), competitive inhibition by dietary protein digestion products often restricts drug transport and triggers unpredictable motor fluctuations (8, 71). High-protein meals increase circulating plasma LNAA levels, thereby reducing central levodopa influx and abruptly terminating antiparkinsonian efficacy without necessarily altering peripheral drug concentrations (45). To mitigate these competitive dynamics, clinical strategies such as low-protein diets or protein redistribution diets (PRDs)—where dietary protein is restricted until the evening—significantly increase daytime levodopa bioavailability, lower circulating diurnal LNAAs, and extend clinical “on” time (72). Beyond amino acid competition, meal ingestion timing strongly governs oral levodopa disposition; consuming meals concurrently or shortly before dosing delays gastric emptying and prolongs the time to peak plasma concentration ( $T_{\max}$ ), leading to reduced maximum plasma concentration ( $C_{\max}$ ) and lower total area under the curve ( AUC ) (47). Non-protein dietary constituents also contribute variably to pharmacokinetic outcomes; while coffee, dietary fiber, and vitamin C have been observed to favorably support levodopa bioavailability, specific botanical substances like fresh banana juice markedly impair its systemic exposure (73). Conversely, macronutrient manipulations such as the ketogenic diet demonstrate minimal effect on plasma levodopa kinetics, underscoring that competitive amino acid inhibition and gastric transit remain the predominant dietary determinants of levodopa efficacy. The competitive transport kinetics across intestinal and blood–brain barrier LAT1 carriers, along with dietary influences on gastric motility and hepatic biotransformation, are schematically detailed in Figure 7. Consequently, optimizing dietary composition, enforcing protein redistribution, and timing meals appropriately relative to drug administration represent essential non-pharmacological interventions to stabilize levodopa pharmacokinetics and enhance therapeutic outcomes in advanced PD (74).

**Figure 7:**
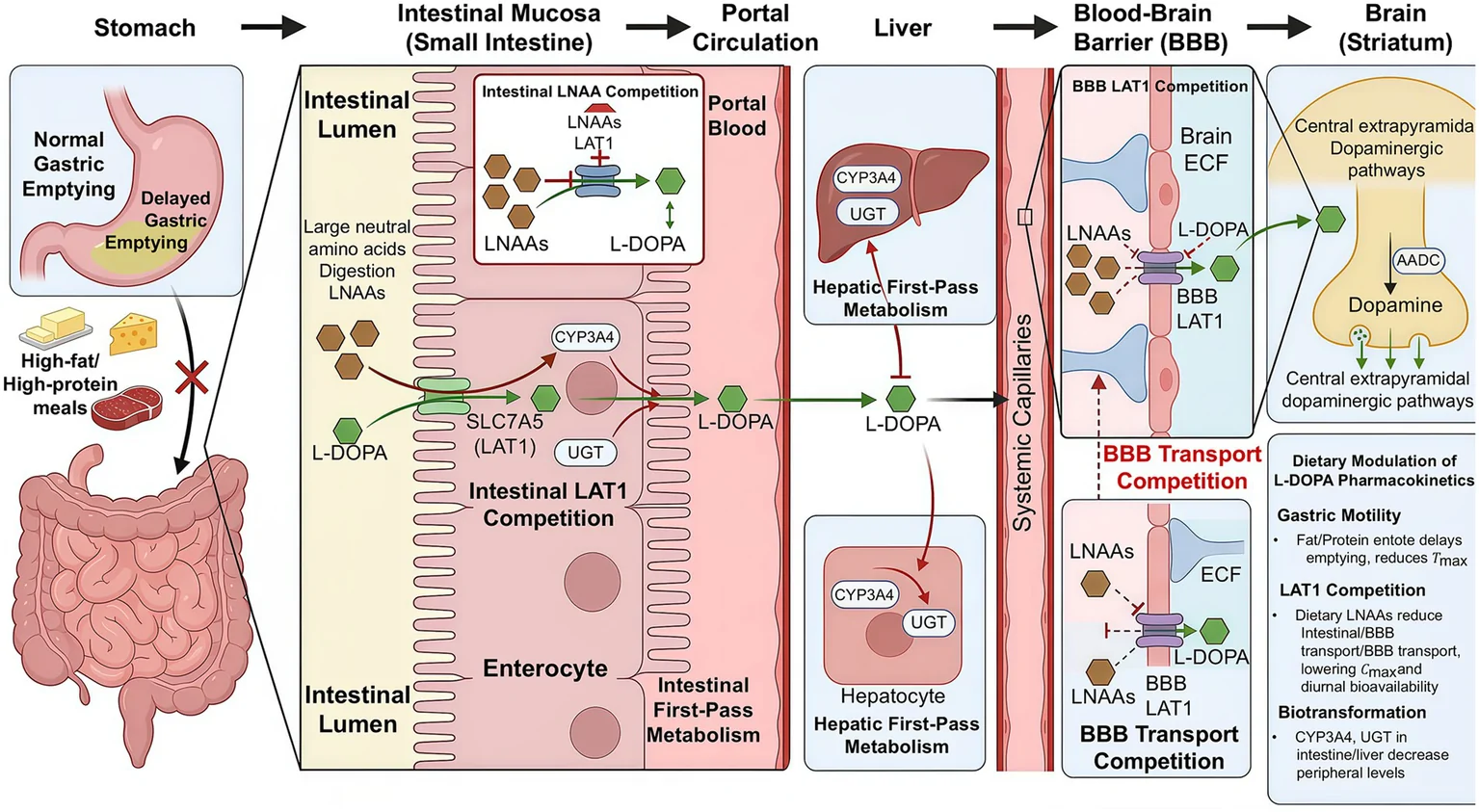
Molecular mechanisms of dietary-mediated levodopa transport competition and pharmacokinetic modulation. This diagram provides a high-resolution view of the competitive transport kinetics and presystemic biotransformation pathways governing levodopa bioavailability. Because levodopa is structurally analogous to endogenous amino acids, it must obligatorily utilize the saturable LAT1 transport system to traverse both the intestinal epithelium and the blood–brain barrier. High-protein meals trigger a rapid influx of LNAAs into the systemic circulation, competitively excluding levodopa from central influx and abruptly terminating antiparkinsonian efficacy. Furthermore, concurrent food ingestion physically delays the time to reach maximum concentration (Tmax) and suppresses the maximum plasma concentration (Cmax), slowing the onset of the clinical response. These competitive dynamics necessitate strategic interventions, such as protein redistribution diets (PRDs) that restrict daytime protein intake, to lower circulating diurnal LNAAs and rescue therapeutic “on” times.

### 4.2 Regulatory effects of dietary factors on neural function and disease progression

Dietary factors exert profound regulatory effects on central nervous system plasticity and disease progression, establishing nutrition as a pivotal modifiable determinant of brain health in neurodegenerative disorders such as PD (75). Overall diet and specific nutritional choices directly modulate fundamental molecular mechanisms—including synaptic plasticity, neurogenesis, and energy metabolism—by providing key substrates and driving neurotrophic signaling pathways, such as brain-derived neurotrophic factor (BDNF) (76). Unhealthy dietary patterns, particularly high-calorie regimes, high-sugar diets, and excessive consumption of saturated or trans-fats, accelerate neurodegenerative cascades by promoting neuroinflammation, exacerbating intracellular oxidative stress, impairing blood–brain barrier integrity, and fostering insulin resistance (77, 78) Conversely, therapeutic dietary approaches such as calorie restriction, intermittent fasting, and Mediterranean-DASH Intervention for Neurodegenerative Delay (MIND) diets demonstrate robust neuroprotective efficacy (79). These health-promoting regimens enhance neuronal resilience and mitigate neuropathology through multifactorial pathways, including the activation of anti-inflammatory cascades, reduction of reactive oxygen species (ROS), suppression of pathological protein aggregation (e.g., amyloid-beta and tau deposition), and the modulation of the hypothalamic–pituitary–adrenal (HPA) axis. Furthermore, targeted immunonutrition involving bioactive compounds—such as omega-3 polyunsaturated fatty acids, polyphenols, B-complex vitamins, and antioxidants—plays a crucial role in maintaining neuronal membrane integrity and regulating neurotransmitter synthesis (80). Beyond direct cellular actions, diet acts as the primary driver of the gut-brain axis; dietary fibers and specific nutrients select for beneficial intestinal microflora, generating microbial metabolites that preserve immune barrier function and reduce systemic inflammation, thereby offering a multifaceted non-invasive strategy to slow disease progression and optimize neural functional outcomes (81).

### 4.3 Diet-mediated disruption of the microbiota-Gut-brain Axis in PD treatment

The microbiota–gut–brain axis (MGBA) operates as a complex, bidirectional communication network that integrates neural, immune, neuroendocrine, and metabolic pathways to maintain central nervous system (CNS) homeostasis and modulate neurodegenerative disease trajectories (82, 83). In PD, pathological alterations in dietary habits and eating behavior patterns directly shape the gut microbial architecture, triggering dysbiosis characterized by a loss of anti-inflammatory, short-chain fatty acid (SCFA)–producing taxa and an overgrowth of pro-inflammatory pathobionts (84). This microbial imbalance compromises gut mucosal barrier integrity and increases intestinal permeability, facilitating the systemic dissemination of lipopolysaccharides and microbial metabolites that provoke chronic low-grade neuroinflammation, microglial activation, and blood–brain barrier dysfunction (85, 86). Furthermore, retrograde transport of preformed α -synuclein aggregates via the vagus nerve—a core neural pathway of the MGBA—directly connects gut dysregulation to the progressive loss of dopaminergic neurons in the substantia nigra, supporting a “gut-first” hypothesis in PD pathogenesis (87). Concurrently, altered microbial metabolism disrupts the synthesis of key neuroactive molecules, such as γ -aminobutyric acid (GABA), tryptophan derivatives, and secondary bile acids acting through farnesoid X receptor (FXR) and Takeda G protein-coupled receptor 5 (TGR5) pathways (88). These neuroactive shifts impair central appetite and satiety signaling while exacerbating hypothalamic–pituitary–adrenal (HPA) axis dysregulation and oxidative stress (89). Beyond driving intrinsic neurodegeneration, MGBA dysregulation heavily impairs the therapeutic efficacy and bioavailability of antiparkinsonian medications; specifically, erratic gastrointestinal transit and altered local bacterial enzymatic activity hinder levodopa absorption and alter peripheral drug metabolism (90). Consequently, targeted therapeutic strategies aimed at restoring MGBA homeostasis—including precision dietary modulation, probiotics, prebiotics, and fecal microbiota transplantation (FMT)—represent highly promising non-invasive interventions to suppress neuroinflammation, stabilize drug pharmacokinetics, and optimize clinical outcomes in PD (91). A summary of the multidimensional mechanisms—spanning pharmacokinetics, neural plasticity, and the microbiota–gut–brain axis—by which dietary factors modulate drug efficacy and disease progression is provided in Table 4. Figure 8 illustrates the microbiota–gut–brain axis pathways, highlighting gut microbial levodopa degradation, mucosal barrier permeability, and retrograde neuroinflammatory signaling that collectively govern medication bioavailability and neurodegeneration.

**Table 4:** Multidimensional biological mechanisms underlying dietary modulation of drug efficacy and disease progression in PD.

Mechanism level	Core Pathway/Physiological process	Key modulatory dietary factors	Specific impact on drugs/disease outcome	Representative clinical intervention strategies	References
Pharmacokinetic (PK) Regulation	LNAA Competition: Competition for LAT1 transporters at the intestine and BBB Gastric Emptying Rate: Gastric motility and intestinal absorption timing	Dietary protein intake (amino acids from digestion) High-fat, heavy, or bulky meals	Reduces levodopa transport across the blood–brain barrier, precipitating sudden “OFF” fluctuations Delays $T_{\max}$ and lowers $C_{\max}$ , slowing down the onset of levodopa clinical response	Protein Redistribution Diet (PRD): Restrict protein during daytime; consume primarily at dinner Administer levodopa 30–60 min before meals or 2 h after meals	(8, 85–90)
Neural Plasticity & Stress Response	BDNF/Synaptic Plasticity: BDNF signaling and neurogenesis promotion Oxidative Stress & Inflammation: ROS generation and microglial activation	High-sugar/high-fat diets (inhibit); Polyphenols & Omega-3 s (promote) Western diet (exacerbates); Calorie restriction/Ketogenic diets (mitigate)	Modulates dopaminergic neuronal survival, synaptic resilience, and central responsiveness to drugs Directly influences the rate of progressive dopaminergic neuronal degeneration in the substantia nigra	Adopt the MIND diet; supplement with dietary polyphenols and neuroprotective lipids Implement mild calorie restriction; increase intake of dietary antioxidants (Vitamins C & E)	(91, 92, 94, 96, 97)
Microbiota–Gut–Brain Axis (MGBA)	Barrier Integrity & Metabolites: SCFA production vs. LPS translocation Direct Bacterial Degradation: Bacterial enzymes (e.g., *Enterococcus faecalis* tdc )	High dietary fiber (boosts SCFAs); High fat (increases LPS) Bacterial Degradation: Bacterial enzymes (e.g., *Enterococcus faecalis* tdc ) Gut dysbiosis/Altered microbial composition	Dictates intestinal permeability, local drug breakdown, and systemic low-grade neuroinflammation Gut bacteria prematurely metabolize peripheral levodopa into dopamine, drastically reducing brain delivery	Administer prebiotics, probiotics, and high-fiber dietary regimens Target gut microbiota balance via precision nutrition or FMT to minimize peripheral drug loss	(98–100, 101–106)

**Figure 8:**
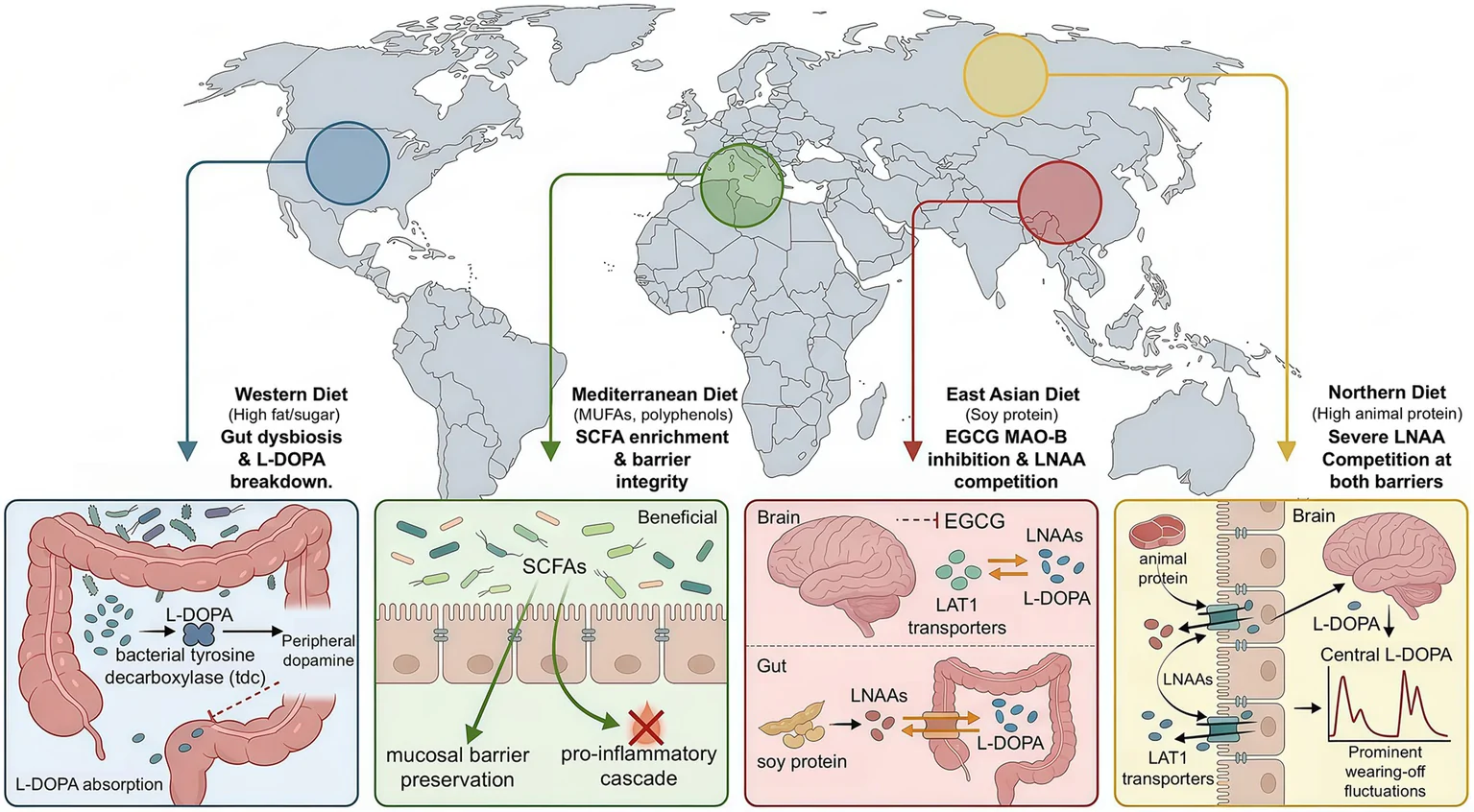
Microbiota–gut–brain axis dynamics and neuroinflammatory signaling in PD drug response. This figure maps the sequential pathological cascade linking diet-induced microbial imbalance to progressive neurodegeneration and drug failure. An unhealthy dietary matrix triggers severe dysbiosis, characterized by the depletion of SCFA-producing taxa and the overgrowth of pathogenic strains. This shift directly degrades the intestinal mucosal barrier, increasing permeability and allowing microbial antigens to infiltrate systemic circulation. This systemic breach facilitates the retrograde transport of preformed α-synuclein aggregates via the vagus nerve directly to the central nervous system, supporting a “gut-first” pathogenic trajectory. Once within the brain, these signals trigger the chronic, abnormal activation of microglia and the generation of reactive oxygen species (ROS), amplifying neuroinflammation, compromising blood–brain barrier integrity, and ultimately accelerating the degeneration of dopaminergic neurons in the substantia nigra pars compacta.

## 5 Clinical translation and dietary management strategies for PD pharmacotherapy

### 5.1 Individualized dietary guidance strategies

Implementing individualized dietary guidance strategies represents a vital paradigm shift to optimize therapeutic efficacy and enhance medication management in clinical populations (92). Rather than relying on generalized population recommendations that often yield inconsistent outcomes, precision nutrition leverages multiomics datasets—including genetic profiles, gut microbiota composition, metabolic biomarkers, and specific lifestyle patterns—to customize dietary recommendations (93). Advanced technologies such as artificial intelligence, machine learning, and smart wearable systems are increasingly integrated into clinical care to analyze real-time phenotypic and physiological data, allowing clinicians to predict individual metabolic responses to specific nutrient timing and food combinations (94). Beyond algorithm-driven decision support, successful individualization requires structured dietary education and continuous behavioral support, such as live coaching and personalized meal frameworks, which significantly improve long-term dietary compliance and patient-reported quality of life (95). Clinical trials across complex chronic conditions demonstrate that tailored dietary interventions are superior to conventional, generalized advice in improving clinical biomarkers, glycemic control, and overall metabolic health (96). Consequently, translating these individualized strategies into PD management could allow clinicians to align specific dietary habits with individual drug absorption characteristics, mitigating pharmacokinetic interactions and maximizing overall therapeutic responses (92).

### 5.2 Optimization of medication administration time and meal timing

Synchronizing medication administration schedules with meal events and circadian biology represents a critical, underutilized strategy for maximizing therapeutic efficacy and minimizing adverse reactions (97). Pharmacokinetic and pharmacodynamic parameters are intrinsically modulated by the circadian clock, as endogenous daily rhythms govern gastrointestinal motility, gastric pH, hepatic metabolism, and renal excretion. Consequently, administering oral formulations without accounting for drug-food interactions or biological timing often leads to suboptimal clinical outcomes; observational clinical data reveal that improper timing relative to meals occurs in up to 15.7% of general oral drug administrations. In conditions requiring precise drug delivery, aligning dosage schedules with meal timing is vital to prevent competitive intragastric interactions and physical incompatibilities (98, 99). For instance, establishing targeted pre-prandial windows or “no-food” intervals ensures proper gastric emptying and prevents nutrient-induced alterations in drug absorption profiles (99). Therefore, implementing precise chronotherapeutic dosing schedules and educating patients on the strategic timing of drug intake relative to meals provides a non-invasive, cost-effective framework to optimize therapeutic response and ensure patient safety (97, 100).

### 5.3 Nutritional support and comprehensive disease management

Integrated nutritional care has emerged as a cornerstone in the holistic, long-term therapeutic strategy for complex, progressive disorders, transitioning from a supportive role to a recognized first-line intervention that directly impacts clinical outcomes (105). Disease-related malnutrition significantly exacerbates systemic complications, accelerates motor and non-motor decline, and increases hospitalization rates, thereby placing a substantial economic burden on healthcare systems. In neurodegenerative pathology, multifactorial mechanisms—including gastrointestinal dysmotility, altered energy expenditure, and progressive dysphagia—frequently impair intake and nutrient absorption, which directly interferes with pharmacotherapeutic optimization. Addressing these metabolic and physical barriers through early, tailored dietary interventions and personalized nutritional support can reduce systemic inflammation, optimize therapeutic efficacy, and lower complication risks (101, 102). Consequently, multidisciplinary clinical management—combining early nutritional screening, physician-guided care, and specialized dietary planning—is essential to preserve patient autonomy, maximize drug responsiveness, and enhance overall quality of life (105, 103).

### 5.4 Development of precision nutrition therapy

The transition from standard dietary guidelines to precision nutrition therapy represents a paradigm shift in managing chronic neurodegenerative conditions like PD. Traditional population-based recommendations rely on average requirements with safety margins, yet individual nutritional and therapeutic demands vary substantially due to distinct genetic predispositions, metabolic profiles, and physical activity levels (104). By integrating multi-omics technologies—such as genomics, transcriptomics, metabolomics, and metagenomics—precision nutrition evaluates inter-individual variability in response to nutrients and medications, moving beyond a “one-size-fits-all” framework to design tailored dietary interventions (105). In the context of neurological and brain disorders, optimizing therapy requires the accurate assessment of individual metabolic readiness, dietary substrate utilization, and specific disease-state dynamics, such as the adaptation of targeted ketogenic therapies or specialized protein-timing strategies to improve therapeutic efficacy (106, 107). Furthermore, advanced computational tools, machine learning algorithms, and real-time digital monitoring devices are crucial for automating the integration of continuous phenotypic data, gut microbiome profiles, and nutrient interactions (107). Ultimately, this data-driven framework allows clinicians to dynamically refine macronutrient, micronutrient, and supplement delivery, thereby preventing adverse nutrient-drug interactions, maximizing levodopa bioavailability, and optimizing clinical outcomes for PD patients (104, 108). A comprehensive translational ecosystem integrating multi-omics profiling, chronotherapeutic meal synchrony, and personalized protein-redistribution strategies for optimizing PD pharmacotherapy is presented in Figure 9.

**Figure 9:**
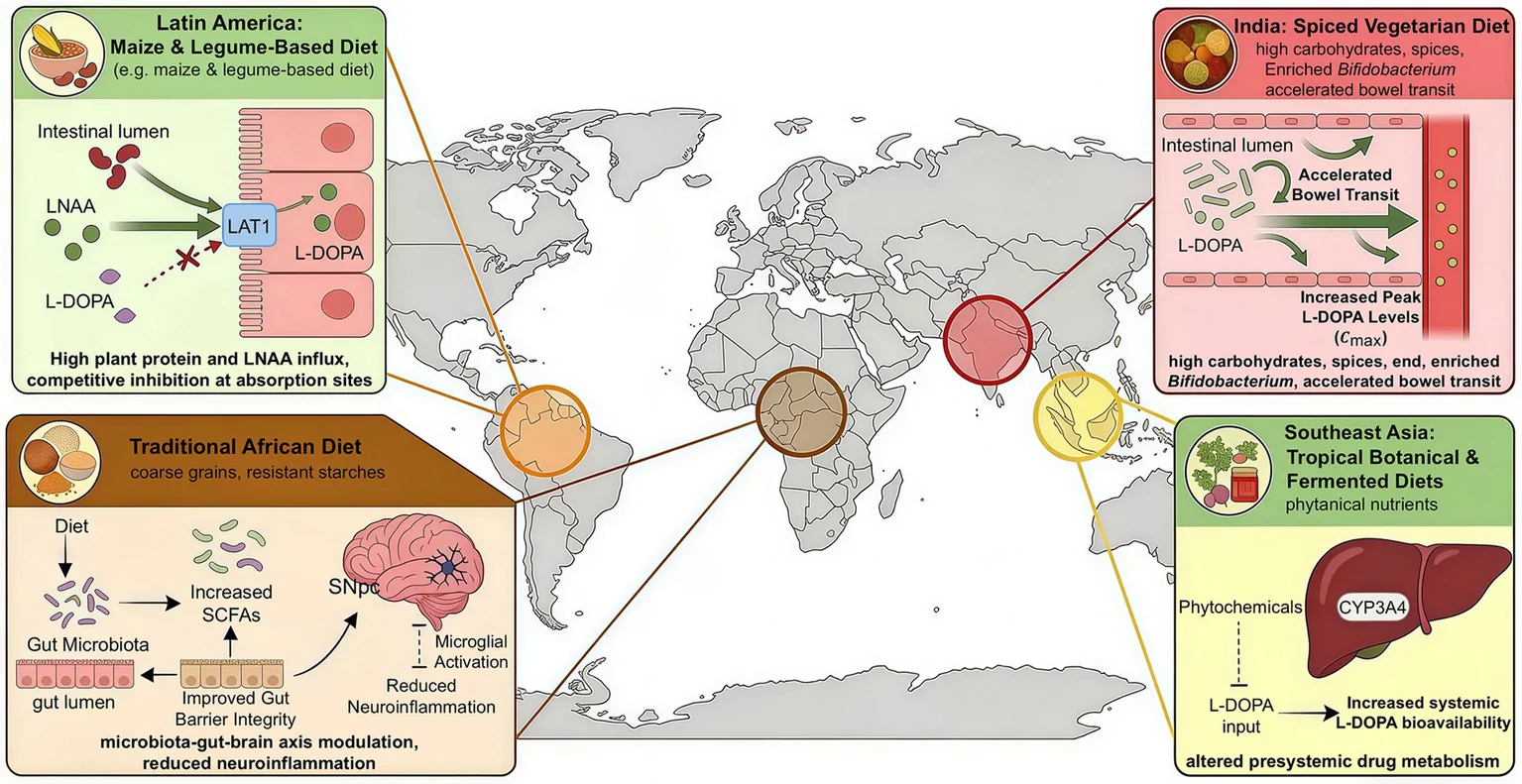
Translational continuum and precision nutrition care ecosystem for personalized PD management. This comprehensive schematic illustrates the transition from generalized dietary guidelines to algorithm-driven precision nutrition therapy. The ecosystem relies on the continuous integration of multi-omics profiling—including patient-specific genomics, transcriptomics, metabolomics, and gut microbiome diversity—to accurately predict inter-individual metabolic responses and drug-nutrient interactions. Central to this framework is the implementation of chronotherapeutic meal scheduling, which strategically synchronizes medication administration with circadian biology, targeted pre-prandial windows, and personalized protein-redistribution strategies. Augmented by smart wearable systems and machine learning algorithms, this data-driven, multidisciplinary clinical support model allows for dynamic refinement of macronutrient delivery, thereby minimizing motor fluctuations, preventing adverse enzymatic interactions, and maximizing holistic therapeutic outcomes.

## 6 Conclusion

Current pharmacological management of PD faces substantial clinical limitations—including motor response oscillations, erratic gastrointestinal absorption, and inter-individual pharmacokinetic variability—which are heavily modulated by intrinsic biological mechanisms and extrinsic dietary factors. A systematic synthesis reveals that dietary patterns and eating behaviors exert multi-dimensional regulatory effects on antiparkinsonian medication efficacy: Western dietary habits, binge eating, and erratic meal timing exacerbate gastrointestinal dysbiosis, delay gastric emptying, alter drug-metabolizing enzymes (such as CYP3A4 and UGT), and promote systemic neuroinflammation; conversely, fiber-rich Eastern/Japanese diets and Mediterranean/MIND patterns preserve gut mucosal integrity and neuronal plasticity. Mechanistically, these outcomes are driven by competitive inhibition between dietary large neutral amino acids and levodopa at intestinal and blood–brain barrier transporters, nutrient-induced kinetic shifts, metabolic-stress pathways, and microbiota–gut–brain axis interactions—including gut dysbiosis, vagal α -synuclein transport, and direct bacterial enzymatic degradation of peripheral levodopa. Consequently, transitioning toward precision nutrition therapy—utilizing multi-omics, protein-redistribution diets, chronotherapeutic meal-dosing synchrony, and comprehensive nutritional support—provides an essential non-pharmacological strategy to stabilize drug absorption, minimize motor fluctuations, mitigate disease progression, and optimize holistic clinical care in PD.

## Glossary

3-OMD: 3-O-methyldopa
A2AR: Adenosine A2A receptor
AUC: Area under the curve
BBB: Blood–brain barrier
BDNF: Brain-derived neurotrophic factor
Cmax: Maximum plasma concentration
CNS: Central nervous system
COMT: Catechol-O-methyltransferase
CYP: Cytochrome P450
DRA: Dopamine receptor agonist
EGCG: Epigallocatechin-3-gallate
FMT: Fecal microbiota transplantation
GABA: Gamma-aminobutyric acid
HPA: Hypothalamic–pituitary–adrenal
ICD: Impulse control disorder
LAT1: L-type amino acid transporter 1
LNAA: Large neutral amino acid
LPS: Lipopolysaccharide
MAO-B: Monoamine oxidase-B
MeDi: Mediterranean diet
MGBA: Microbiota–gut–brain axis
MIND: Mediterranean-DASH Intervention for Neurodegenerative Delay
MUFA: Monounsaturated fatty acid
OATP: Organic anion transporting polypeptide
PD: Parkinson’s disease
PRD: Protein-redistribution diet
ROS: Reactive oxygen species
SCFA: Short-chain fatty acid
tdc: Tyrosine decarboxylase
Tmax: Time to reach maximum concentration
TMAO: Trimethylamine N-oxide
UGT: UDP-glucuronosyltransferase

## References

[1] Grotewold N Albin RL. Update: descriptive epidemiology of Parkinson disease. Parkinsonism Relat Disord. (2024) 120:106000. doi: 10.1016/j.parkreldis.2024.106000, 38233324 PMC10922566

[2] Tysnes O-B Storstein A. Epidemiology of Parkinson’s disease. J Neural Transm. (2017) 124:901–5. doi: 10.1007/s00702-017-1686-y, 28150045

[3] Willis AW Roberts E Beck JC Fiske B Ross W Savica R . Incidence of Parkinson disease in North America. NPJ Parkinsons Dis. (2022) 8:170. doi: 10.1038/s41531-022-00410-y, 36522332 PMC9755252

[4] Li A Cha A Huang M Dhami S Pemminati S. A review of the recent advances in the pharmacological management of Parkinson’s disease. Cureus. (2025) 17:e93123. doi: 10.7759/cureus.93123 PMC12551797 41141087

[5] Arabia G. Pharmacological treatment of Parkinson’s disease. BMC Geriatr. (2010) 10:L30. doi: 10.1186/1471-2318-10-S1-L30

[6] Müller T. Update on the present and future pharmacologic treatment of Parkinson’s disease. Neurol Ther. (2025) 14:1769–81. doi: 10.1007/s40120-025-00800-3, 40679671 PMC12450148

[7] Boelens Keun JT Arnoldussen IA Vriend C van de Rest O. Dietary approaches to improve efficacy and control side effects of levodopa therapy in Parkinson’s disease: a systematic review. Adv Nutr. (2021) 12:2265–87. doi: 10.1093/advances/nmab060, 34113965 PMC8634393

[8] Rusch C Flanagan R Suh H Subramanian I. To restrict or not to restrict? Practical considerations for optimizing dietary protein interactions on levodopa absorption in Parkinson’s disease. npj Parkinson Dis. (2023) 9:98. doi: 10.1038/s41531-023-00541-w, 37355689 PMC10290638

[9] Menozzi E Schapira AHV. The Gut microbiota in Parkinson disease: interactions with drugs and potential for therapeutic applications. CNS Drugs. (2024) 38:315–31. doi: 10.1007/s40263-024-01073-4, 38570412 PMC11026199

[10] Tambasco N Romoli M Calabresi P. Levodopa in Parkinson’s disease: current status and future developments. Curr Neuropharmacol. (2018) 16:1239–52. doi: 10.2174/1570159X15666170510143821, 28494719 PMC6187751

[11] Contin M Martinelli P. Pharmacokinetics of levodopa. J Neurol. (2010) 257:S253–61. doi: 10.1007/s00415-010-5728-8, 21080186

[12] Calabresi P Ghiglieri V Mazzocchetti P Corbelli I Picconi B. Levodopa-induced plasticity: a double-edged sword in Parkinson’s disease? Philos Trans R Soc B Biol Sci. (2015) 370:20140184. doi: 10.1098/rstb.2014.0184 PMC4455753 26009763

[13] Calabresi P Di Filippo M Ghiglieri V Picconi B. Molecular mechanisms underlying levodopa-induced dyskinesia. Mov Disord. (2008) 23:S570–9. doi: 10.1002/mds.22019 18781674

[14] Demailly A Moreau C Devos D. Effectiveness of continuous dopaminergic therapies in Parkinson’s disease: a review of L-DOPA pharmacokinetics/pharmacodynamics. J Parkinsons Dis. (2024) 14:925–39. doi: 10.3233/JPD-230372 PMC11307025 38848195

[15] Kwon DK Kwatra M Wang J Ko HS. Levodopa-induced dyskinesia in Parkinson’s disease: pathogenesis and emerging treatment strategies. Cells. (2022) 11:3736. doi: 10.3390/cells11233736, 36496996 PMC9736114

[16] Schapira AHV. The clinical relevance of levodopa toxicity in the treatment of Parkinson’s disease. Mov Disord. (2008) 23:S515–20. doi: 10.1002/mds.22146 18781678

[17] Stocchi F. Dopamine agonists in Parkinson’s disease. CNS Drugs. (1998) 10:159–70.

[18] Tan Y-Y Jenner P Chen S-D. Monoamine oxidase-B inhibitors for the treatment of Parkinson’s disease: past, present, and future. J Parkinsons Dis. (2022) 12:477–93. doi: 10.3233/JPD-212976, 34957948 PMC8925102

[19] Alborghetti M Bianchini E De Carolis L Galli S Pontieri FE Rinaldi D. Type-B monoamine oxidase inhibitors in neurological diseases: clinical applications based on preclinical findings. Neural Regen Res. (2023) 19:16–21. doi: 10.4103/1673-5374.375299, 37488838 PMC10479837

[20] Regensburger M Ip CW Kohl Z Schrader C Urban PP Kassubek J . Clinical benefit of MAO-B and COMT inhibition in Parkinson’s disease: practical considerations. J Neural Transm. (2023) 130:847–61. doi: 10.1007/s00702-023-02623-8, 36964457 PMC10199833

[21] Alcalay RN Gu Y Mejia-Santana H Cote L Marder KS Scarmeas N. The association between Mediterranean diet adherence and Parkinson’s disease. Mov Disord. (2012) 27:771–4. doi: 10.1002/mds.24918, 22314772 PMC3349773

[22] Maraki MI Yannakoulia M Xiromerisiou G Stefanis L Charisis S Giagkou N . Mediterranean diet is associated with a lower probability of prodromal Parkinson’s disease and risk for Parkinson’s disease/dementia with Lewy bodies: a longitudinal study. Eur J Neurol. (2023) 30:934–42. doi: 10.1111/ene.15698, 36692092

[23] Naoi M Maruyama W Shamoto-Nagai M. Neuroprotective function of rasagiline and selegiline, inhibitors of type B monoamine oxidase, and role of monoamine oxidases in Synucleinopathies. Int J Mol Sci. (2022) 23:11059. doi: 10.3390/ijms231911059, 36232361 PMC9570229

[24] Finberg JPM. Inhibitors of MAO-B and COMT: their effects on brain dopamine levels and uses in Parkinson’s disease. J Neural Transm. (2019) 126:433–48. doi: 10.1007/s00702-018-1952-7, 30386930

[25] Jenner P. An overview of adenosine A2A receptor antagonists in Parkinson’s disease. Int Rev Neurobiol. (2014) 119:71–86. doi: 10.1016/B978-0-12-801022-8.00003-9, 25175961

[26] Pourcher E Huot P. Adenosine 2A receptor antagonists for the treatment of motor symptoms in Parkinson’s disease. Mov Disord Clin Pract. (2015) 2:331–40. doi: 10.1002/mdc3.12187 PMC6178748 30363540

[27] López-Taboada I González-Pardo H Conejo NM. Western diet: implications for brain function and behavior. Front Psychol. (2020) 11:564413. doi: 10.3389/fpsyg.2020.564413 PMC7719696 33329193

[28] Alfonsetti M Castelli V d’Angelo M. Are we what we eat? Impact of diet on the Gut–brain Axis in Parkinson’s disease. Nutrients. (2022) 14:380–19. doi: 10.3390/nu14020380, 35057561 PMC8780419

[29] Beckers M Bloem BR Verbeek MM. Mechanisms of peripheral levodopa resistance in Parkinson’s disease. NPJ Parkinsons Dis. (2022) 8:56. doi: 10.1038/s41531-022-00321-y, 35546556 PMC9095610

[30] Ali A Ahamad R Aqil M Mir MI Biswas AS. "Pharmacokinetics". In: Gupta S, editor. IIP Series, vol. 3. Chikmagalur, Karnataka, India: Iterative International Publishers (IIP) (2023). p. 78–88.

[31] Wu K Kwon SH Zhou X Fuller C Wang X Vadgama J . Overcoming challenges in small-molecule drug bioavailability: a review of key factors and approaches. Int J Mol Sci. (2024) 25:13121. doi: 10.3390/ijms252313121, 39684832 PMC11642056

[32] Thummel KE Lin YS. Sources of interindividual variability. Methods Mol Biol. (2014) 1113:363–415. doi: 10.1007/978-1-62703-758-7_17, 24523121

[33] Shook BC Jackson PF. Adenosine A2A receptor antagonists and Parkinson’s disease. ACS Chem Neurosci. (2011) 2:555–67. doi: 10.1021/cn2000537, 22860156 PMC3369712

[34] Liu J Lu Y Tang M Shao F Yang D Chen S . Fucoxanthin prevents long-term administration l-DOPA-induced neurotoxicity through the ERK/JNK-c-Jun system in 6-OHDA-lesioned mice and PC12 cells. Mar Drugs. (2022) 20:245. doi: 10.3390/md20040245 PMC9025159 35447917

[35] Paknahad Z Sheklabadi E Moravejolahkami AR Chitsaz A Hassanzadeh A. The effects of Mediterranean diet on severity of disease and serum Total antioxidant capacity (TAC) in patients with Parkinson’s disease: a single center, randomized controlled trial. Nutr Neurosci. (2022) 25:313–20. doi: 10.1080/1028415X.2020.1751509, 32319358

[36] Paknahad Z Sheklabadi E Derakhshan Y Bagherniya M Chitsaz A. The effect of the Mediterranean diet on cognitive function in patients with Parkinson’s disease: a randomized clinical controlled trial. Complement Ther Med. (2020) 50:102366. doi: 10.1016/j.ctim.2020.102366, 32444045

[37] Chu C Yu L Chen W Tian F Zhai Q. Dietary patterns affect Parkinson’s disease via the microbiota-gut-brain axis. Trends Food Sci Technol. (2021) 116:90–101. doi: 10.1016/j.tifs.2021.07.004

[38] Li T Wu J Zhou SY Li MA Zhao LP Wang A . Neuroprotective effects of time-restricted feeding combined with different protein sources in MPTP-induced Parkinson’s disease mice model and its modulatory impact on gut microbiota metabolism. Adv Sci. (2026):e16502. doi: 10.1002/advs.202516502 PMC13045433 41588858

[39] Azam F Mohamed N Alhussen F. Molecular interaction studies of green tea catechins as multitarget drug candidates for the treatment of Parkinson’s disease: computational and structural insights. Netw Comput Neural Syst. (2015) 26:97–115. doi: 10.3109/0954898X.2016.1146416 27030558

[40] Hu X Yu L Li Y Li X Zhao Y Xiong L . Piperine improves levodopa availability in the 6-OHDA-lesioned rat model of Parkinson’s disease by suppressing gut bacterial tyrosine decarboxylase. CNS Neurosci Ther. (2023) 30:e14383. doi: 10.1111/cns.14383 PMC10848080 37528534

[41] Nie W Li L. Synergistic neuroprotective effects of traditional Chinese medicine and levodopa in Parkinson’s disease: a behavioral and pharmacokinetic study. J Vis Exp. (2025). doi: 10.3791/69260 41182962

[42] Huang L Wei W Huang X Li X Liu H Gui L . High-fat diets enhance and delay ursodeoxycholic acid absorption but elevate circulating hydrophobic bile salts. Front Pharmacol. (2023) 14:1168144. doi: 10.3389/fphar.2023.1168144, 37138846 PMC10149867

[43] Velchik MG Reynolds JC Alavi A. The effect of meal energy content on gastric emptying. J Nucl Med. (1989) 30:1106–10. 2738691

[44] Tsui JK Ross S Poulin K Douglas J Postnikoff D Calne S . The effect of dietary protein on the efficacy of L-dopa: a double-blind study. Neurology. (1989) 39:549–9. doi: 10.1212/WNL.39.4.549, 2494567

[45] Carter JH Nutt JG Woodward WR Hatcher LF Trotman TL. Amount and distribution of dietary protein affects clinical response to levodopa in Parkinson’s disease. Neurology. (1989) 39:552–2. doi: 10.1212/WNL.39.4.552, 2648188

[46] Bracco F Malesani R Saladini M Battistin L. Protein redistribution diet and antiparkinsonian response to levodopa. Eur Neurol. (2008) 31:68–71. doi: 10.1159/000116649, 2044618

[47] Contin M Riva R Martinelli P Albani F Baruzzi A. Effect of meal timing on the kinetic-dynamic profile of levodopa/carbidopa controlled is release in parkinsonian patients. Eur J Clin Pharmacol. (1998) 54:303–8. doi: 10.1007/s002280050464, 9696954

[48] O’Keefe SJD Li JV Lahti L Ou J Carbonero F Mohammed K . Fat, fiber and cancer risk in African Americans and rural Africans. Nat Commun. (2015) 6:6342. doi: 10.1038/ncomms7342 PMC4415091 25919227

[49] Isibor PO Akinduti PA Aworunse OS Oyewale JO Oshamika O Ugboko HU . Significance of African diets in biotherapeutic modulation of the gut microbiome. Bioinforma Biol Insights. (2021) 15:11779322211012697. doi: 10.1177/11779322211012697 PMC8107938 33994782

[50] Wang Q Luo Y Ray Chaudhuri K Reynolds R Tan EK Pettersson S. The role of gut dysbiosis in Parkinson’s disease: mechanistic insights and therapeutic options. Brain J Neurol. (2021) 144:2571–93. doi: 10.1093/brain/awab156, 33856024

[51] Phillips RD. Starchy legumes in human nutrition, health and culture. Plant Foods Hum Nutr. (1993) 44:195–211. doi: 10.1007/BF01088314, 8295859

[52] Ernandes M La Guardia M Giammanco S. Maize based diets and possible neurobehavioural after-effects among some populations in the world. Hum Evol. (1996) 11:67–77. doi: 10.1007/bf02456990

[53] Graham GG Lembcke J Morales E. Post-prandial plasma aminograms in the assessment of protein quality for young children: maize and grain amaranth, alone and combined. Eur J Clin Nutr. (1990) 44:35–43. 2354691

[54] Tomova A Bukovsky I Rembert E Yonas W Alwarith J Barnard ND . The effects of vegetarian and vegan diets on Gut microbiota. Front Nutr. (2019) 6:47–7. doi: 10.3389/fnut.2019.00047, 31058160 PMC6478664

[55] Dahl WJ Mendoza DR Lambert JM. "Diet, nutrients and the microbiome". In: Sun J, editor. Progress in Molecular Biology and Translational Science, vol. 171. Cambridge, MA, USA: Academic Press (Elsevier) (2020). p. 237–63. 10.1016/bs.pmbts.2020.04.006 32475524

[56] Meiners F Ortega-Matienzo A Fuellen G Barrantes I. Gut microbiome-mediated health effects of fiber and polyphenol-rich dietary interventions. Front Nutr. (2025) 12:1647740. doi: 10.3389/fnut.2025.1647740, 40948860 PMC12425962

[57] Randeni N Bordiga M Xu B. A comprehensive review of the triangular relationship among diet–gut microbiota–inflammation. Int J Mol Sci. (2024) 25:9366–6. doi: 10.3390/ijms25179366 PMC11394685 39273314

[58] Verdegaal AA Goodman AL. Integrating the gut microbiome and pharmacology. Sci Transl Med. (2024) 16:eadg8357. doi: 10.1126/scitranslmed.adg8357 PMC12121898 38295186

[59] Harris RZ Jang GR Tsunoda SM. Dietary effects on drug metabolism and transport. Clin Pharm. (2003) 42:1071–88. doi: 10.2165/00003088-200342130-00001, 14531721

[60] Yu Y Miller R Groth SW. A literature review of dopamine in binge eating. J Eat Disord. (2022) 10:11. doi: 10.1186/s40337-022-00531-y, 35090565 PMC8796589

[61] Alagha M Al-Alam F Saroufine K Elias L Klaimi M Nabbout G . Binge eating disorder and metabolic syndrome: shared mechanisms and clinical implications. Healthcare. (2025) 13:482. doi: 10.3390/healthcare13050482, 40077044 PMC11898466

[62] Coppens J Bentea E Bayliss J Demuyser T Walrave L Albertini G . Caloric restriction protects against lactacystin-induced degeneration of dopamine neurons independent of the ghrelin receptor. Int J Mol Sci. (2017) 18:558. doi: 10.3390/ijms18030558 PMC5372574 28273852

[63] Polaka S Rajpoot K Tekade M Sharma MC Tekade RK. "Chapter 11 - food–drug interactions and their implications on oral drug bioavailability". In: Tekade RK, editor. Pharmacokinetics and Toxicokinetic Considerations, vol. 2. Cambridge, MA, USA: Academic Press (2022). p. 263–89.

[64] Mattson MP Allison DB Fontana L Harvie M Longo VD Malaisse WJ . Meal frequency and timing in health and disease. Proc Natl Acad Sci USA. (2014) 111:16647–53. doi: 10.1073/pnas.1413965111, 25404320 PMC4250148

[65] Yu G Wu D-N Gong Y Li G-F Zhou H-H. Conflicting meal recommendations for oral oncology drugs: pose risks to patient care? Eur J Clin Pharmacol. (2018) 74:833–42. doi: 10.1007/s00228-018-2439-z 29536134

[66] Fleisher D Li C Zhou Y Pao LH Karim A. Drug, meal and formulation interactions influencing drug absorption after oral administration. Clinical implications. Clin Pharmacokinet. (1999) 36:233–54. doi: 10.2165/00003088-199936030-00004, 10223170

[67] Fagerberg P Klingelhoefer L Bottai M Langlet B Kyritsis K Rotter E . Lower energy intake among advanced vs. early Parkinson’s disease patients and healthy controls in a clinical lunch setting: a cross-sectional study. Nutrients. (2020) 12:2109. doi: 10.3390/nu12072109, 32708668 PMC7400863

[68] Athlin E Norberg A Axelsson K Möller A Nordström G. Aberrant eating behavior in elderly parkinsonian patients with and without dementia: analysis of video-recorded meals. Res Nurs Health. (1989) 12:41–51. 10.1002/nur.4770120107 2522233

[69] Voon V Fox SH. Medication-related impulse control and repetitive Behaviors in Parkinson disease. Arch Neurol. (2007) 64:1089–96. doi: 10.1001/archneur.64.8.1089, 17698698

[70] Rosenberg P Herishanu Y Beilin B. Increased appetite (bulimia) in Parkinson’s disease. J Am Geriatr Soc. (1977) 25:277–8. doi: 10.1111/j.1532-5415.1977.tb00415.x, 266536

[71] van Kessel SP El Aidy S. Contributions of gut bacteria and diet to drug pharmacokinetics in the treatment of Parkinson’s disease. Front Neurol. (2019) 10:1087. doi: 10.3389/fneur.2019.01087, 31681153 PMC6803777

[72] Juncos JL Fabbrini G Mouradian MM Serrati C Chase TN. Dietary influences on the antiparkinsonian response to levodopa. Arch Neurol. (1987) 44:1003–5. doi: 10.1001/archneur.1987.00520220009006, 3632369

[73] Fernandez N Garcia JJ Diez MJ Sahagun AM Díez R Sierra M. Effects of dietary factors on levodopa pharmacokinetics. Expert Opin Drug Metab Toxicol. (2010) 6:633–42. doi: 10.1517/17425251003674364, 20384552

[74] Di Mavungu JD Diana Di Mavungu J Malysheva SV Sanders M Larionova D Robbens J . Development and validation of a new LC–MS/MS method for the simultaneous determination of six major ergot alkaloids and their corresponding epimers. Application to some food and feed commodities. Food Chem. (2012) 135:292–303. doi: 10.1016/j.foodchem.2012.04.098

[75] Gomez-Pinilla F Gomez AG. The influence of dietary factors in central nervous system plasticity and injury recovery. PM&R. (2011) 3:S111–6. doi: 10.1016/j.pmrj.2011.03.001, 21703566 PMC3258094

[76] Gómez-Pinilla F. Brain foods: the effects of nutrients on brain function. Nat Rev Neurosci. (2008) 9:568–78. doi: 10.1038/nrn2421, 18568016 PMC2805706

[77] Wen B Han X Gong J Wang P Sun W Xu C . Nutrition: a non-negligible factor in the pathogenesis and treatment of Alzheimer’s disease. Alzheimers Dement. (2025) 21:e14547. doi: 10.1002/alz.14547 PMC11863745 39868840

[78] Sowińska M Gut M Adamczyk A Bezara D Ważny M Gancarz A . The impact of diet on neurodegenerative diseases. J Educ Health Sport. (2026) 88:69542. doi: 10.12775/JEHS.2026.88.69542

[79] Lobo F Haase J Brandhorst S. The effects of dietary interventions on brain aging and neurological diseases. Nutrients. (2022) 14:5086. doi: 10.3390/nu14235086, 36501116 PMC9740746

[80] Naureen Z Dhuli K Medori MC Caruso P Manganotti P Chiurazzi P . Dietary supplements in neurological diseases and brain aging. J Prev Med Hyg. (2022) 63:E174–88. doi: 10.15167/2421-4248/jpmh2022.63.2S3.2759 PMC9710403 36479494

[81] Frausto DM Forsyth CB Keshavarzian A Voigt RM. Dietary regulation of Gut-brain Axis in Alzheimer’s disease: importance of microbiota metabolites. Front Neurosci. (2021) 15:736814. doi: 10.3389/fnins.2021.736814, 34867153 PMC8639879

[82] Stanimirov B Đanić M Pavlović N Zaklan D Lazarević S Mikov M . Gut-brain Axis and bile acid Signaling: linking microbial metabolism to brain function and metabolic regulation. Int J Mol Sci. (2025) 26:12167. doi: 10.3390/ijms262412167, 41465592 PMC12733927

[83] Yang J Song X Yan S Li Q Yang W. The gut microbiota influences neurodegenerative diseases through the gut-brain axis: molecular mechanisms and effects on immune function. Front Immunol. (2026) 16:1739329. doi: 10.3389/fimmu.2025.1739329, 41601624 PMC12832290

[84] Tang Y Zhang Y Chen C Cao Y Wang Q Tang C. Gut microbiota: a new window for the prevention and treatment of neuropsychiatric disease. J Cent Nerv Syst Dis. (2025) 17:11795735251322450. doi: 10.1177/11795735251322450, 39989718 PMC11846125

[85] Chen C Wang G Li D Zhang F. Microbiota–gut–brain axis in neurodegenerative diseases: molecular mechanisms and therapeutic targets. Mol Biomed. (2025) 6:64. doi: 10.1186/s43556-025-00307-1, 40952592 PMC12436269

[86] Faysal M Zehravi M Sutradhar B al Amin M Shanmugarajan TS Arjun UVNV . The microbiota-Gut-brain connection: a new horizon in neurological and neuropsychiatric disorders. CNS Neurosci Ther. (2025) 31:e70593. doi: 10.1111/cns.70593, 40908772 PMC12411739

[87] Doenyas C Clarke G Cserjési R. Gut–brain axis and neuropsychiatric health: recent advances. Sci Rep. (2025) 15:3415. doi: 10.1038/s41598-025-86858-3, 39870727 PMC11772745

[88] Li S Liu M Han Y Liu C Cao S Cui Y . Gut microbiota-derived gamma-aminobutyric acid improves host appetite by inhibiting satiety hormone secretion. mSystems. (2024) 9:e0101524. doi: 10.1128/msystems.01015-24, 39315776 PMC11495008

[89] Asadi A Shadab Mehr N Mohamadi MH Shokri F Heidary M Sadeghifard N . Obesity and gut–microbiota–brain axis: a narrative review. J Clin Lab Anal. (2022) 36:e24420. doi: 10.1002/jcla.24420, 35421277 PMC9102524

[90] Yadav H Jaldhi Bhardwaj R Anamika Bakshi A Gupta S . Unveiling the role of gut-brain axis in regulating neurodegenerative diseases: a comprehensive review. Life Sci. (2023) 330:122022. doi: 10.1016/j.lfs.2023.122022 37579835

[91] Salam MA. Microbiota–gut–brain axis: a new horizon in mapping disease, health and beyond. TAJ J Teach Assoc. (2025) 38:167–81. doi: 10.70818/taj.v038i03.0359

[92] Antwi J. Precision nutrition to improve risk factors of obesity and type 2 diabetes. Curr Nutr Rep. (2023) 12:679–94. doi: 10.1007/s13668-023-00491-y, 37610590 PMC10766837

[93] Mundt C Yusufoğlu B Kudenko D Mertoğlu K Esatbeyoglu T. AI-driven personalized nutrition: integrating omics, ethics, and digital health. Mol Nutr Food Res. (2025) 69:e70293. doi: 10.1002/mnfr.70293, 41163310 PMC12700061

[94] Varshney N. Personalized dietary recommendations using machine learning: a comprehensive review. 2023 International Conference on Artificial Intelligence for Innovations in Healthcare Industries (ICAIIHI) 1–6 (2023)

[95] Couto VDCS Jaime PC Louzada ML d C. Translating the Brazilian dietary guidelines into clinical practice: innovative strategies for healthcare professionals. Arch Endocrinol Metab. (2025) 69:e240142. doi: 10.20945/2359-4292-2024-0142, 39936829 PMC11834862

[96] Omura Y Murakami K Matoba K Nishimura R Sasaki S. Effects of individualized dietary advice compared with conventional dietary advice for adults with type 2 diabetes: a randomized controlled trial. Nutr Metab Cardiovasc Dis. (2022) 32:1035–44. doi: 10.1016/j.numecd.2021.11.006, 35115208

[97] Kaşkal M Sevim M Ülker G Keleş C Bebitoğlu BT. The clinical impact of chronopharmacology on current medicine. Naunyn Schmiedeberg's Arch Pharmacol. (2025) 398:6179–91. doi: 10.1007/s00210-025-03788-7, 39792169 PMC12125046

[98] Grannell L. When should I take my medicines? Aust Prescr. (2019) 42:86–9. doi: 10.18773/austprescr.2019.025, 31363305 PMC6594844

[99] Camilleri M. Drug-resin drug interactions in patients with delayed gastric emptying: what is optimal time window for drug administration? Neurogastroenterol Motil. (2016) 28:1268–71. doi: 10.1111/nmo.12823, 26987693 PMC4956542

[100] Cheng L Wong H. Food effects on Oral drug absorption: application of physiologically-based pharmacokinetic Modeling as a predictive tool. Pharmaceutics. (2020) 12:672. doi: 10.3390/pharmaceutics12070672, 32708881 PMC7408216

[101] Gafiyatullina R Binda J. Nutritional interventions in chronic disease management: evidence-based approaches for improved patient outcomes. Scientific J Bielsko. (2024) 28:155–65. doi: 10.19192/wsfip.sj4.2024.22

[102] Denlinger KL Carr P Waddell DC Mack J. A recyclable, metal-free mechanochemical approach for the oxidation of alcohols to carboxylic acids. Molecules. (2020) 25:364. doi: 10.3390/molecules25020364, 31963148 PMC7024246

[103] Volkert D Beck AM Cederholm T Cruz-Jentoft A Goisser S Hooper L . ESPEN guideline on clinical nutrition and hydration in geriatrics. Clin Nutr. (2019) 38:10–47. doi: 10.1016/j.clnu.2018.05.024, 30005900

[104] Berciano S Figueiredo J Brisbois TD Alford S Koecher K Eckhouse S . Precision nutrition: maintaining scientific integrity while realizing market potential. Front Nutr. (2022) 9:979665. doi: 10.3389/fnut.2022.979665, 36118748 PMC9481417

[105] Livingstone KM Ramos-Lopez O Pérusse L Kato H Ordovas JM Martínez JA. Precision nutrition: a review of current approaches and future endeavors. Trends Food Sci Technol. (2022) 128:253–64. doi: 10.1016/j.tifs.2022.08.017

[106] Stoppe C Hill A Christopher KB Kristof AS. Toward precision in nutrition therapy. Crit Care Med. (2025) 53:e429–40. doi: 10.1097/CCM.0000000000006537, 39688452 PMC11801434

[107] Kirk D Catal C Tekinerdogan B. Precision nutrition: a systematic literature review. Comput Biol Med. (2021) 133:104365. doi: 10.1016/j.compbiomed.2021.104365, 33866251

[108] Fang L Zhuang Y Zhang M Yang D Zhang R Peng J . Dietary and metabolic reprogramming alleviates neurodegeneration: a review of mechanisms and clinical implications. Front Nutr. (2026) 13:1706597. doi: 10.3389/fnut.2026.1706597, 41693955 PMC12893954

[109] Guo Z Ersoz A Butterfield DA Mattson MP. Beneficial effects of dietary restriction on cerebral cortical synaptic terminals. J Neurochem. (2000) 75:314–20. doi: 10.1046/j.1471-4159.2000.0750314.x, 10854276

