# Supplementary material for: Effects of eating behavior patterns and dietary habits on the therapeutic efficacy of Parkinson’s disease medications: from phenomena, mechanisms to clinical applications
Source: Front Nutr. 2026 Oct 19;13:1969279. doi: 10.3389/fnut.2026.1969279 (PMC13725110; doi:10.3389/fnut.2026.1969279)
Supplement: Supplementary file 1 [file Supplementary_file_1.docx]

**Table S1. Clinical studies directly evaluating dietary or meal-related effects on pharmacological treatment in Parkinson’s disease**

| **Dietary pattern / intervention** | **Medication involved** | **Study design / population** | **Main findings** | **Clinical implications** | **Reference** |
| --- | --- | --- | --- | --- | --- |
| Very low daytime protein / high-protein comparison | Levodopa/carbidopa | Clinical dietary intervention; 11 PD patients with ON–OFF fluctuations | Near-zero daytime protein increased levodopa sensitivity and reduced fluctuations; 8 patients required a 41% reduction in total levodopa dose. High-protein intake was associated with marked bradykinesia. | Protein restriction can markedly potentiate levodopa response in selected protein-sensitive fluctuators; dose adjustment and dyskinesia monitoring may be required. | Pincus & Barry, 1987. Arch Neurol 44:270–272. doi:10.1001/archneur.1987.00520150026014 |
| High-protein formula vs recommended protein intake | Levodopa/carbidopa | Experimental dietary study; 6 PD patients with motor fluctuations | A high-protein formula increased plasma LNAAs and prematurely terminated the antiparkinsonian response; protein intake at the recommended daily allowance had no clinically appreciable effect. | Avoid assuming that all protein intake is detrimental; excess protein may be most relevant in susceptible patients with advanced fluctuations. | Juncos et al., 1987. Arch Neurol 44:1003–1005. doi:10.1001/archneur.1987.00520220009006 |
| High-protein vs low-protein diet | Levodopa/carbidopa ± other stable PD medications | Randomized double-blind dietary crossover; 10 PD patients, each diet for 1 week | Motor performance was significantly better on the low-protein diet, with fewer OFF hours; differences were not explained by plasma levodopa concentrations. | Supports a clinically important central LNAA competition mechanism and a trial of lower protein intake in protein-sensitive motor fluctuations. | Tsui et al., 1989. Neurology 39:549–552. doi:10.1212/WNL.39.4.549 |
| High protein, low protein evenly distributed, and protein-redistribution diet | Levodopa | Within-subject comparison; 5 PD patients with motor fluctuations | Mean ON time was 51% with 1.6 g/kg protein, 67% with 0.8 g/kg evenly distributed protein, and 77% with 0.8 g/kg protein restricted to the evening. Mean plasma levodopa did not differ materially. | Both total protein load and its distribution across the day can modify clinical levodopa response. | Carter et al., 1989. Neurology 39:552–556. doi:10.1212/WNL.39.4.552 |
| Balanced carbohydrate:protein meal (5:1) vs high-protein/low-carbohydrate and low-protein/high-carbohydrate meals | Carbidopa/levodopa | Meal-composition study; 9 PD patients | The balanced meal produced less variability in plasma LNAAs and superior motor performance than the other meal compositions, despite levodopa increasing after all meals. | Meal composition, not only absolute protein restriction, may help stabilize levodopa:LNAA competition while maintaining nutritional adequacy. | Berry et al., 1991. Neurology 41:1295–1297. doi:10.1212/WNL.41.8.1295 |
| Protein-redistribution diet (protein largely deferred to evening) | Levodopa | Prospective dietary intervention; 16 PD patients with daily response fluctuations | Five patients had a clear significant reduction in diurnal motor fluctuations, and the overall group tended toward a more constant levodopa response. | PRD may be a practical adjunct for selected patients with levodopa-related fluctuations, although response is heterogeneous. | Bracco et al., 1991. Eur Neurol 31:68–71. doi:10.1159/000116649 |
| Meal 30 min before vs 2 h after controlled-release levodopa/carbidopa | Controlled-release levodopa/carbidopa 200/50 mg | Randomized within-subject crossover; 8 PD patients on chronic levodopa | Taking levodopa after the meal almost doubled the time of initial plasma appearance and Tmax, reduced the 6-h AUC by about 24%, delayed motor-response onset, and shortened response duration. | Timing oral levodopa relative to meals can materially alter both pharmacokinetics and clinical response, even with controlled-release formulations. | Contin et al., 1998. Eur J Clin Pharmacol 54:303–308. doi:10.1007/s002280050464 |
| Usual protein intake / clinically reported protein interaction | Levodopa | Retrospective clinical-record analysis; 1,037 PD patients | Protein-related fluctuations were reported in 5.9% of levodopa-treated patients and 12.4% of those with motor fluctuations; earlier wearing-off and reduced dose efficacy were common. Dietary modification was also associated with weight loss in some patients. | Protein sensitivity is clinically important but affects a subset rather than all levodopa-treated patients; nutritional monitoring is essential. | Virmani et al., 2016. J Clin Mov Disord 3:8. doi:10.1186/s40734-016-0036-9 |
| Ketogenic diet (approximately 80% fat, 15% protein, 5% carbohydrate) | Levodopa | Small human pharmacokinetic study; 4 PD participants followed the diet for 12 weeks | No significant change in levodopa Cmax, Tmax, or AUC was observed compared with baseline. | Not all major dietary-pattern changes alter levodopa pharmacokinetics; ketogenic diet should not be presented as a proven enhancer of levodopa exposure. | Elbarbry et al., 2019. Biomed Chromatogr 33:e4382. doi:10.1002/bmc.4382 |
